# Novel scheme for defining the clinical implications of *TP53* mutations in myeloid neoplasia

**DOI:** 10.1186/s13045-023-01480-y

**Published:** 2023-08-03

**Authors:** Waled Bahaj, Tariq Kewan, Carmelo Gurnari, Arda Durmaz, Ben Ponvilawan, Ishani Pandit, Yasuo Kubota, Olisaemeka D. Ogbue, Misam Zawit, Yazan Madanat, Taha Bat, Suresh K. Balasubramanian, Hussein Awada, Ramsha Ahmed, Minako Mori, Manja Meggendorfer, Torsten Haferlach, Valeria Visconte, Jaroslaw P. Maciejewski

**Affiliations:** 1 Department of Translational Hematology and Oncology Research, Taussig Cancer Institute, Cleveland Clinic, 9620 Carnegie Ave N Building, Building NE6-250, Cleveland, OH 44106 USA; 2 Division of Medical Oncology & Hematology, School of Medicine, University of Louisville, Louisville, KY USA; 3 Division of Hematology & Oncology, Yale School of Medicine, New Haven, CT USA; 4 Department of Biomedicine and Prevention, Ph.D. in Immunology, Molecular Medicine and Applied Biotechnology, University of Rome Tor Vergata, Rome, Italy; 5 Department of Internal Medicine, Division of Hematology and Oncology, University of Texas Southwestern Medical Center, Dallas, TX USA; 6 Department of Hematology and Oncology, Wayne State University, Detroit, MI USA; 7 MLL Munich Leukemia Laboratory, Munich, Germany

**Keywords:** *TP53* mutations, Allelic inactivation, Myeloid neoplasia, Next-generation sequencing, Single-cell DNA sequencing

## Abstract

**Background:**

*TP53* mutations (*TP53*^*MT*^) occur in diverse genomic configurations. Particularly, biallelic inactivation is associated with poor overall survival in cancer. Lesions affecting only one allele might not be directly leukemogenic, questioning the presence of cryptic biallelic subclones in cases with dismal prognosis.

**Methods:**

We have collected clinical and molecular data of 7400 patients with myeloid neoplasms and applied a novel model by identifying an optimal VAF cutoff using a statistically robust strategy of sampling-based regression on survival data to accurately classify the *TP53* allelic configuration and assess prognosis more precisely.

**Results:**

Overall, *TP53*^*MT*^ were found in 1010 patients. Following the traditional criteria, 36% of the cases were classified as single hits, while 64% exhibited double hits genomic configuration. Using a newly developed molecular algorithm, we found that 579 (57%) patients had unequivocally biallelic, 239 (24%) likely contained biallelic, and 192 (19%) had most likely monoallelic *TP53*^*MT*^. Interestingly, our method was able to upstage 192 out of 352 (54.5%) traditionally single hit lesions into a probable biallelic category. Such classification was further substantiated by a survival-based model built after re-categorization. Among cases traditionally considered monoallelic, the overall survival of those with probable monoallelic mutations was similar to the one of wild-type patients and was better than that of patients with a biallelic configuration. As a result, patients with certain biallelic hits, regardless of the disease subtype (AML or MDS), had a similar prognosis. Similar results were observed when the model was applied to an external cohort. In addition, single-cell DNA studies unveiled the biallelic nature of previously considered monoallelic cases.

**Conclusion:**

Our novel approach more accurately resolves *TP53* genomic configuration and uncovers genetic mosaicism for the use in the clinical setting to improve prognostic evaluation.

**Supplementary Information:**

The online version contains supplementary material available at 10.1186/s13045-023-01480-y.

## Key Points

- Patients with single *TP53* mutations and variant allele frequency more than 23% act biologically like biallelic *TP53* cases.
- Traditionally defined single hits might contain subclones with biallelic inactivation, which negatively influences prognosis.

**Supplementary Information:**

The online version contains supplementary material available at 10.1186/s13045-023-01480-y.

## Introduction

*TP53* is a pivotal tumor suppressor gene (TSG) in cancer, including myeloid neoplasia (MN). *TP53* can be affected by hypomorphic/loss-of-function (LOF) lesions occurring in diverse configurations. In addition to truncated (frameshifts and stop codons), missense mutations in various hotspots may exert a dominant-negative effect [1, 2]. Poor prognosis has been attributed to *TP53* mutations (*TP53*^*MT*^) and loss of heterozygosity (LOH) by 17p deletion (del(17p)) in MN. In particular, this is true for instances as complex karyotype and presumed chemotherapy-related causation [3, 4]. Considering the many variables of *TP53*^*MT*^ topography and configuration, the assessment of the prognostic impact of *TP53* lesions may be challenging [3, 5].

Congenital heterozygous *TP53*^*MT*^ in Li-Fraumeni syndrome (LFS) are the first hits in the oncogenic cascade preceding the evolution of cancers after biallelic inactivation of this gene, a process consistent with a 2-hit theory of recessive TSG inactivation (Knudson’s hypothesis) [6]. Incomplete penetrance in LFS indicates that the residual function of *TP53* is at least partially protective [7]. Monoallelic *TP53*^*MT*^ may also occur in clonal hematopoiesis without overt leukemia [8]. Thus, somatic monoallelic lesions might not be directly leukemogenic if not accompanied by subsequent hits in trans configuration or affecting other genes. In early SNP-arrays studies, we have shown that some patients with *TP53*^*MT*^ may harbor biallelic inactivation by somatic copy-neutral LOH (CN-LOH) or cryptic macro/microdeletions [9]. Presumed monoallelic somatic *TP53*^*MT*^ did not affect survival but biallelic hits did confer an unfavorable prognosis [10, 11]. Furthermore, second *TP53* hits represent the most associated lesions in patients with primary *TP53*^*MT*^ with usually sweeping biallelic subclones, consistent with their functional and clinical impact [12].

The correct assessment of *TP53 *inactivation status in patients with *TP53*^*MT*^ MN in a clinical setting is complicated, as the distinction between two monoallelic hits in a form of subclonal mosaicism vs. true biallelic lesions is not easily possible. Moreover, cases with seemingly monoallelic *TP53*^*MT*^ likely contain cryptic clones with biallelic *TP53* inactivation, but their detection is not possible using traditional sequencing methods. Even after estimating the clonality of del(17p) or uniparental disomy (UPD), such *TP53* configuration analysis is hampered by essential flaws such as the inability to: i) detect and quantify the biallelic fraction in cases with smaller variant allelic frequency (VAF) or ii) prove the presence of subclonal mosaicisms with two different *TP53*^*MT*^ clones using traditional bulk DNA sequencing methods. The accuracy of single-cell sequencing technologies although allowing for such delineation is limited by various technical and feasibility problems. For instance, the use of single-cell DNA analysis is clinically impractical and may not always be conclusive due to allelic dropout and other imprecisions [11]. Similarly, SNP-arrays, while precise, are not sensitive in detecting smaller clones (< 20%) containing deletions or CN-LOH [13]. As a result, small clones possibly constituting a significant portion of occult *TP53* biallelic inactivation are likely to be overseen. [9, 11, 12, 14].

Most previous reports focused on myelodysplastic syndrome (MDS), and secondary acute myeloid leukemia (sAML) have shown that *TP53*^*MT*^ affect the clinical prognostic scoring [4, 11, 15]. Indeed, in the Molecular International Scoring System (IPSS-M) *TP53* lesions are the most impactful on clinical outcomes and the different *TP53* genomic configuration constitutes an important variable [16]. Based on the assumption that biallelic rather than single *TP53* hits are directly leukemogenic, we hypothesized that clinical outcomes such as survival might be used to differentiate cases with biallelic, often cryptic *TP53*^*MT*^ clones (and vice versa). Therefore, we investigated *TP53*^*MT*^ in a fashion agnostic to the subtypes of MN by devising a new rational method able to predict the impact of these mutations on prognosis in real-life scenarios to further improve the current clinical algorithms.

## Materials and methods

### Patient cohort

We have compiled molecular and clinical data of a meta-analytic cohort of 1010 patients with *TP53* alterations, along with 6390 *TP53*^*WT*^ cases. Data were collected from The Cleveland Clinic, (CC, n = 1357), The Munich Leukemia Laboratory (MLL, n = 1962), and publicly available data sets (Memorial Sloan Kettering Cancer Center [11], The Cancer Genome Atlas (TCGA) [17], The BEAT AML master trial, n = 4081 [18] (Additional file 1: Table S1).

### Genetic studies

For the data collected at CC, whole exome sequencing (WES) was performed [19–21] on a subset of samples. Paired tumor and germline DNAs were used for WES. Data were validated using a TruSeq or Nextera platform Custom Amplicon Kit (Illumina, San Diego, CA, USA). The targeted sequencing panel is shown in Additional file 1: Table S2. Variants were annotated using Annovar and filtered using an in-house bioanalytical pipeline [14, 19, 21]. The gene sequencing methods of publicly shared data were previously described [22, 23].

### Statistical analyses

To identify the optimal VAF cutoff able to delineate *TP53*^*MT*^ allelic status, we utilized a statistically robust strategy of subsampling-based regression on survival data. Specifically, using R package randomForestSRC [24], we built a survival model with VAF as a single covariate and minimum node size set to 15. (Further statistical analyses are described in supplementary methods).

## Results

### Clinical characteristics of patients’ cohort

**Fig. 1:**
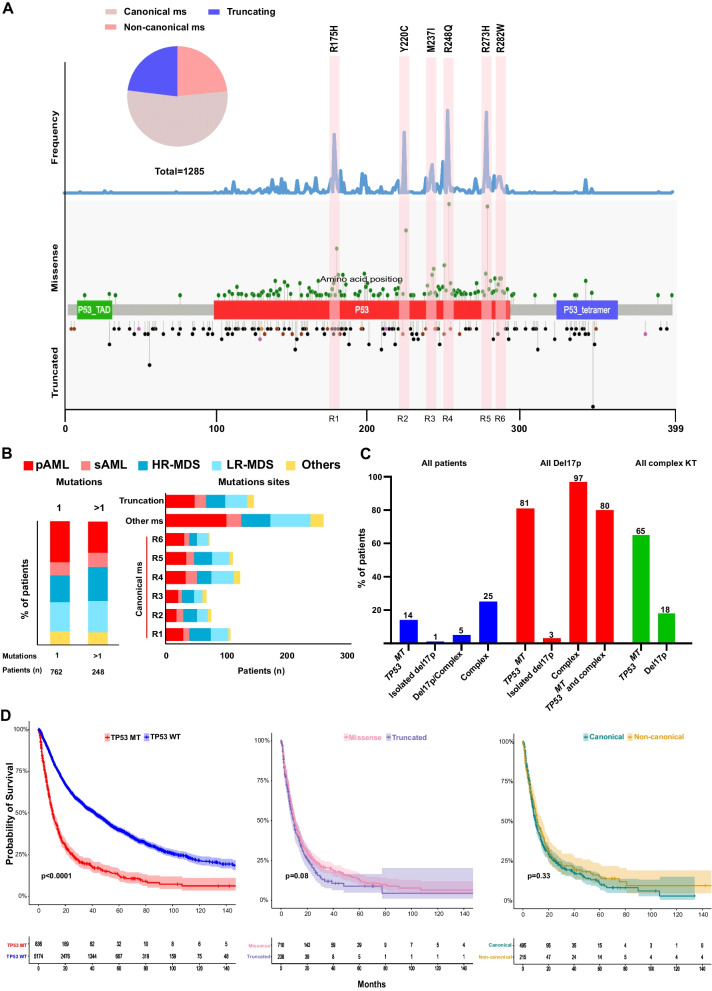
Distribution of *TP53* mutations with hotspot locations and chromosome 17 deletion. **A** Schematic drawing of *TP53* gene showing the location of mutations, the type of mutations, and canonical sites. Frequency of mutations in all cohort is shown in the upper part. Missense and truncated mutations are indicated in the upper and lower part of the gene structure, respectively. **B** Bar graphs showing the frequencies of single (1) and multiple *TP53* mutations (> 1) and canonical missense locations in each disease subtype. **C** Percentage of patients with cytogenetics abnormalities in relation to the *TP53* mutational status. **D** Kaplan–Meier survival curves of patients with *TP53* mutations *vs.* wild type, missense *vs.* truncated mutations, and canonical *vs.* non-canonical missense mutations

We screened a cohort of 7400 patients with MN and found that 1010 (14%) of the patients had 1285 *TP53*^*MT*^ (Figs. 1A, Additional file 1: Figure S1, Table S3). The median age at diagnosis was 70 years (IQR 61–77). Low-risk MDS (LR-MDS), high-risk MDS (HR-MDS), sAML, and primary AML (pAML) were present in 34%, 17%, 4%, and 27%, respectively (Table 1 and Additional file 1: Figure S1). For this study, HR-MDS patients were defined by a blast count of ≥ 5%. The majority of *TP53*^*MT*^ patients harbored complex karyotype at cytogenetic evaluation (704/981; 72%) (Table 1).

**Table 1:** Clinical and cytogenetic characteristics of the study cohort

Characteristics	All patients N (%)	*TP53*^*MT*^ N (%)	*TP53*^*WT*^ N (%)	p-value
Number of patients	7400 (100%)	1010 (14%)	6390 (86%)	
Median age at diagnosis (IQR)	70.3 (61–77)	71 (64–77)	70 (61–77)	0.001
Male gender (%)	58%	53%	59%	0.015
Disease subtypes				
pAML	1985 (26.8%)	316 (31.2%)	1669 (26.1%)	< 0.001
sAML	272 (3.6%)	111 (10.9%)	161 (2.5%)	< 0.001
HR–MDS	1273 (17.2%)	234 (23.1%)	1038 (16.2%)	< 0.001
LR–MDS	2558 (34.6%)	244 (24.1%)	2314 (36.3%)	< 0.001
MDS/MPN	1312 (17.8%)	105 (10.4%)	1208 (18.9%)	< 0.001
Cytogenetic data*				
Normal	2084 (47.8%)	122 (12.4%)	1967 (58.2%)	< 0.001
Complex	1083 (24.8%)	704 (71.7%)	344 (10.1%)	< 0.001
Deletion 5q	211 (4.8%)	95 (9.6%)	116 (3.4%)	< 0.001
Deletion 7	146 (3.3%)	20 (2.0%)	126 (3.7%)	0.009
Deletion 17p	207 (4.7%)	167 (17.0%)	40 (1.1%)	< 0.001
Deletion 20q	93 (2.1%)	8 (0.8%)	85 (2.5%)	0.001
Trisomy 8	252 (5.7%)	36 (3.6%)	216 (6.3%)	0.001
Deletion Y	84 (1.9%)	10 (1.0%)	74 (2.1%)	0.018
Median Hb (IQR)	9.7 (8.5–11.1)	9.1 (8.2–10.2)	9.8 (8.6–11.2)	< 0.001
Median WBC (IQR)	6.8 (3.3–23.6)	5.1 (2.6–16.3)	7.1 (3.4–24.9)	< 0.001
Median platelet (IQR)	105 (50–208)	65 (38–120)	113 (54–220)	< 0.001

^*^Cytogenetics were available for total of 4,358 patients, of these 981 *TP53*^*MT*^ and 3377 *TP53*^*WT*^

*MT* mutation, *WT* wild type, *IQR* interquartile range, *pAML* primary acute myeloid leukemia, *sAML* secondary acute myeloid leukemia, *HR-MDS* high-risk myelodysplastic syndrome, *LR-MDS* low-risk myelodysplastic syndrome, *MDS/MPN* myelodysplastic/myeloproliferative overlap neoplasms, *MPN* myeloproliferative syndrome, *CMML* chronic myelomonocytic leukemia, *Hb* hemoglobin, *WBC* white blood cells

### Distribution of *TP53* mutations and 17p deletions

Of *TP53*^*MT*^ detected, missense mutations were registered in 74% of patients, truncations in 15%, while 11% had concomitant missense and truncated hits (Fig. 1A). Missense mutations were distributed into 6 main sites, including canonical hotspot lesions (defined here as ± 5 amino acids from the most canonical sites, Fig. 1A). Majority of the missense *TP53*^*MT*^ (69%) were detected in the canonical sites (R175H, Y220C, M237I, R248Q, R273H, R282W) [1] (Additional file 1: Figure S2). Notably, we did not observe any differences in the number of *TP53*^*MT*^ nor in the location of the lesions among various MN subtypes (Fig. 1B). Overall, 203 patients had del(17p), of whom 118 (58%) had missense *TP53*^*MT*^, and 17% had truncated *TP53*^*MT*^, 6% of the patients had concurrent missense and truncated *TP53*^*MT*^, while in 19% no mutation was found. Mutations often coincided with deletions of *TP53* locus either as isolated lesions or more often in the context of complex karyotype, wherein *TP53*^*MT*^ was found in 81% of the patients with del(17p) (Fig. 1C). Irrespective of configuration, *TP53*^*MT*^ carriers had worse overall survival (OS) compared to *TP53*^*WT*^ carriers (HR 2.7 [95%CI 2.53–3.02]). No significant differences in OS between truncated and missense *TP53*^*MT*^ or canonical and non-canonical missense *TP53*^*MT*^ were observed (Fig. 1D & B).

### Different *TP53* configurations and disease subtypes impact prognosis

**Fig. 2:**
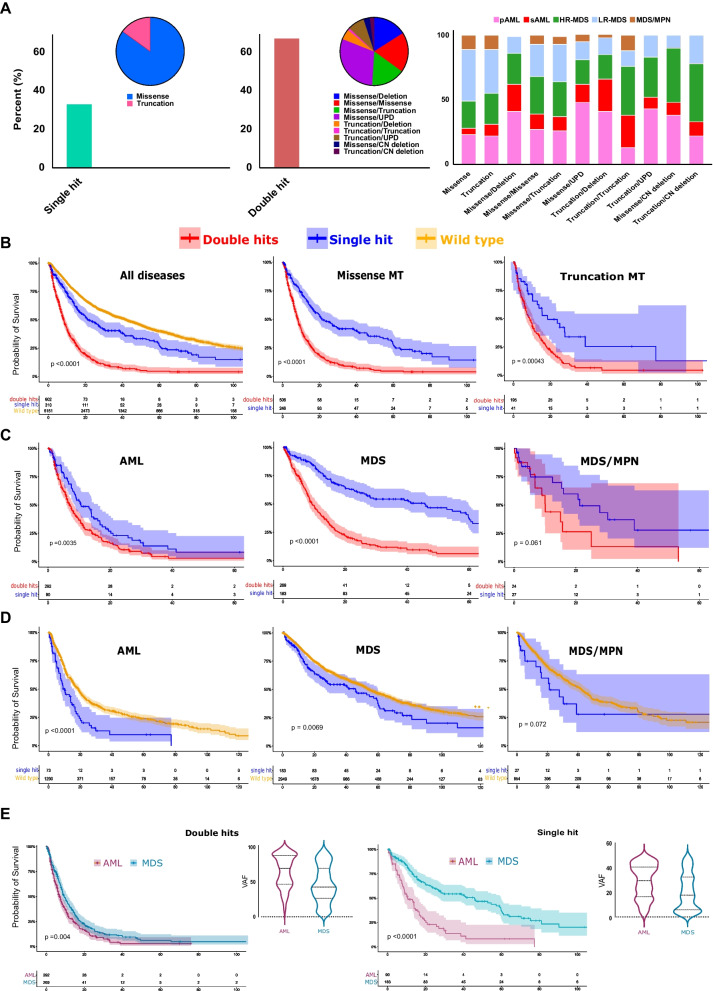
Diverse *TP53* configurations and disease subtypes impact prognosis. **A** patients with single hit (left panel) *vs.* double hits (middle panel) *TP53* lesions and the type of configuration for each hit class and various constellations among different myeloid neoplasms (right panel). **B** Kaplan–Meier survival estimates of patients with *TP53* wild type, single hits, and double hits, all patients (left panel) and cases with missense (middle panel) and truncated mutations (right panel). **C** Kaplan–Meier survival estimates of patients with *TP53* single hits and double hits distributed according to different disease subtypes: AML (left panel), MDS (middle panel), and MDS/MPN (right panel). **D** Kaplan–Meier survival estimates of patients with *TP53* wild type and single hit distributed according to different disease subtypes: AML (left panel), MDS (middle panel), and MDS/MPN (right panel). **E** Kaplan–Meier survival estimates of *TP53* double hits (left panel) and single hit (right panel) in acute myeloid leukemia (AML) *vs.* myelodysplastic syndrome (MDS) in correlation with variant allelic frequency (VAF) for each subtype

Following the traditional definition [10, 11, 25], we categorized the patients into single- and double-*TP53*^*MT*^ hit groups. Briefly, a single *TP53* hit was defined as either: (i) one *TP53* mutation or (ii) isolated 17p deletion, while double *TP53* hits were defined as *TP53*^*MT*^ and (i) another *TP53*^*MT*^ or (ii) 17p deletion or (iii) *TP53* locus UPD. We found that 36% of *TP53*^*MT*^ patients had single hits, while 64% exhibited double hits (Fig. 2A). *TP53* double hits with different configurations were enriched in p/sAML and HR-MDS cases (Fig. 2A, middle and right panels and Additional file 1: Figure S3). Overall, carriers of *TP53* double hits, whether missense and/or truncated had a worse OS than those with single *TP53* hits (HR: 2.5 [2.08–3.02], Fig. 2B. Subgroup analysis according to the underlying disease morphology yielded similar results (*data not shown*). However, the significantly larger difference in OS between single and double *TP53* hits was observed in MDS (HR: 3.1 [2.43–4.09]) compared to AML (HR: 1.5 [1.16–2.04]; Fig. 2C). *TP53*^*MT*^ was also associated with worse OS when we compared *TP53* single hit to *TP53*^*WT*^ in both AML (HR: 1.8 [1.42–2.46]) and MDS (HR: 1.3 [1.08–1.67]) but not in MDS/MPN subtype (Fig. 2C and D). Ultimately, while the OS of AML cases was consistently worse compared to that of MDS cases across all patients, we observed that the magnitude of difference was more significant in the patents with presumed monoallelic *TP53*^*MT*^ (Fig. 2E). Paralleling these findings, such a worse prognosis was also accompanied by a significantly higher VAF of *TP53*^*MT*^ in AML *vs.* MDS in both double hit (64% *vs.* 46.5%, *p* < 0.001) and single hit (median 30 *vs*. 18%, *p* < 0.001; Fig. 2E) lesions.

### A new approach to resolve the dilemma of biallelic ***vs***. monoallelic ***TP53***^***MT***^

**Fig. 3:**
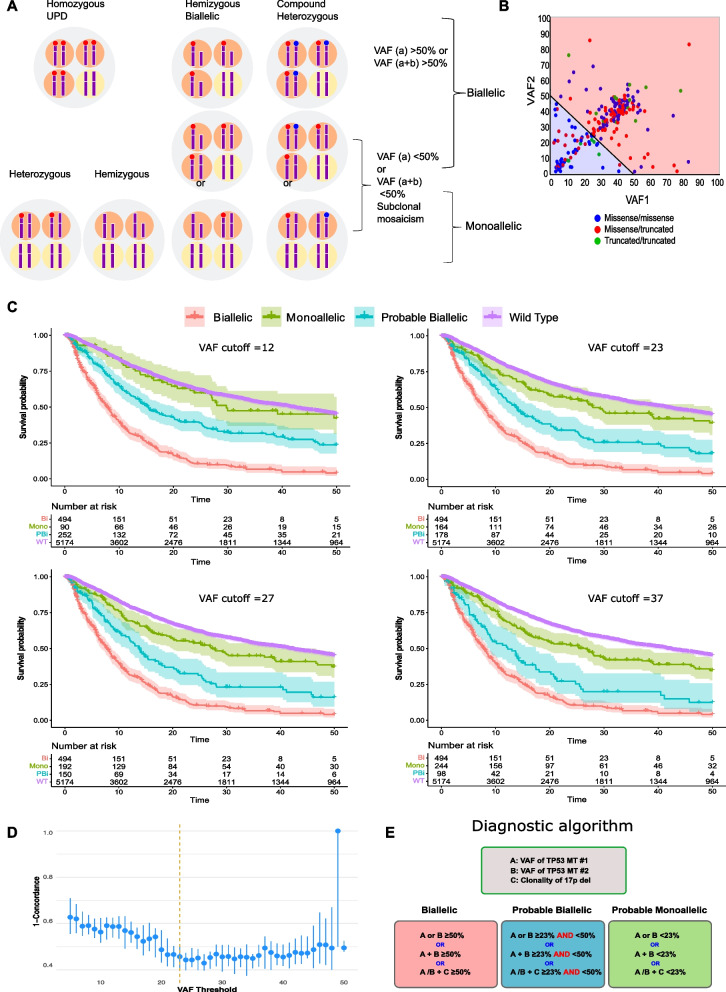
Assessment of allelic status of *TP53* lesions. **A** possibilities of different *TP53* configurations and del(17p) presence resulting in monoallelic *vs.* biallelic lesions. For illustration purpose, four cells scheme is presented. For biallelic mutations, the sum VAF% is more than 50% in hemizygous, homozygous uniparental disomy (UPD) or compound heterozygous configurations. For monoallelic mutations, the sum VAF% is less than 50% in different configurations including heterozygous, hemizygous, or subclonal mosaicism. **B** The variant allele frequency (VAF) of two *TP53* mutations of patients in our cohort was plotted in two colors (red area, obligatory biallelic) with the sum of VAF1 and VAF2 exceeding 50% and (blue area, non-obligatory biallelic) with sum of VAF1 and VAF2 less than 50. **C** Kaplan–Meier survival estimates of obligatory biallelic, probable biallelic, probable monoallelic, and wild-type *TP53* patients after applying different VAF cutoffs based on random forest analysis separating probable monoallelic from probable biallelic mutations. **D** VAF cutoffs cross-validation by randomly splitting the data into test/train sets with %20/%80 ratios and calculating the Harrell’s C-index (Concordance-index) in the test set over 30 runs. A VAF of 23% resulted optimal for separating the monoallelic and biallelic *TP53* mutations. **E** A novel algorithm for the precise classification of *TP53*^*MT*^ into obligatory biallelic, probable biallelic, or probable monoallelic groups

**Fig. 4:**
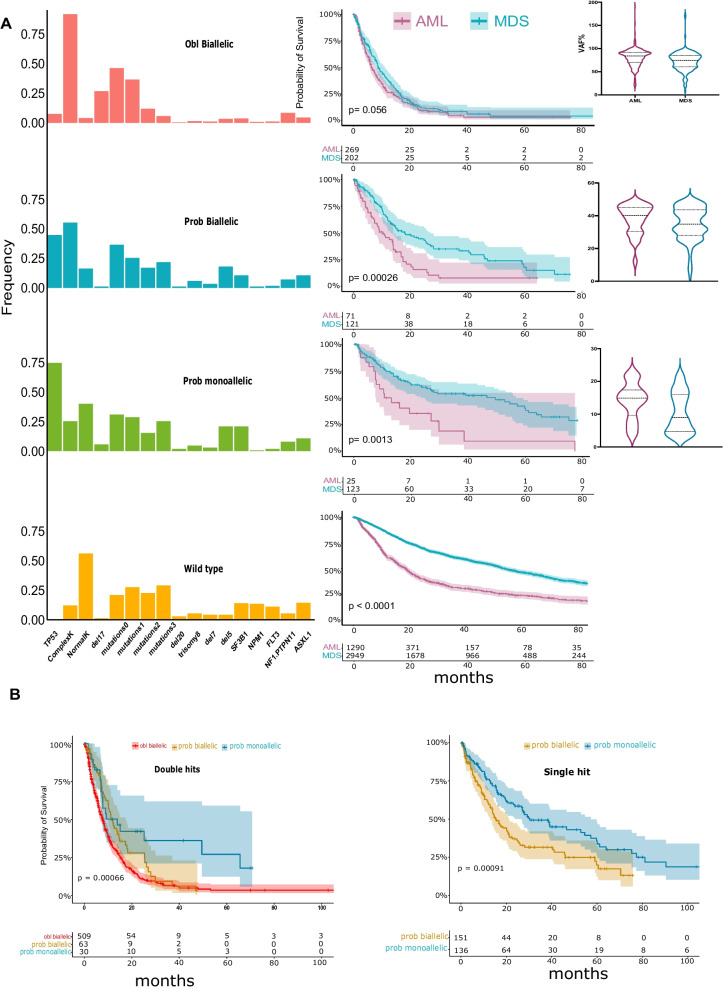
Identifying the probable allelic involvement in relation to clinical and cytogenetic factors. **A** Frequency and importance of number of concurrent somatic mutations and cytogenetic abnormalities with Kaplan–Meier survival estimates comparing acute myeloid leukemia (AML) *vs.* myelodysplastic syndrome (MDS) for each group. Median variant allele frequency (VAF) for AML *vs*. MDS. **B** Kaplan–Meier survival estimates comparing obligatory biallelic, probable biallelic, and probable monoallelic mutations in double hits and single hit groups

To resolve the uncertainty regarding the precise *TP53*^*MT*^ allelic status, we developed a new approach to accurately predict the *TP53* allelic configuration. Based on VAF values available from clinical sequencing, obligatory biallelic *TP53*^*MT*^ were identified among patients with: i) one *TP53* hit and VAF > 50% or ii) two *TP53* hits with a combined VAF > 50% or iii) *TP53*^*MT*^ VAF + clonality of del(17p) > 50% (Fig. 3A, B & Additional file 1: Fig. S6). However, the presence of subclonal mosaicism *vs*. biallelic *TP53* hits (deletion and homozygous mutation) may not be discriminated with one *TP53*^*MT*^ and VAF < 50%. Similarly, patients with ≥ 2 *TP53* hits and a combined VAF < 50% may have subclonal mosaicism of several purely monoallelic or biallelic hits (Additional file 1: Fig. S7). Previously presumed monoallelic cases by traditional classification should be differentiated into those likely true monoallelic *TP53*^*MT*^ and/or those likely containing a subclonal cryptic biallelic *TP53* lesions. Since the presence of biallelic *TP53*^*MT*^ clones is associated with poor OS, we applied a random forest regression analysis, with survival as a surrogate marker for *TP53* allelic status, to fine-tune the VAF cutoff in order to separate the questionable cases into likely monoallelic *vs*. likely biallelic. We cross-validated VAF cutoff values by randomly splitting the data into test/train sets with 20%/80% ratios and calculating the Harrell’s C-index (Concordance) in the test set over 30 runs (Fig. 3D, Additional file 1: Table S4). A VAF cutoff of 23% was found to be optimal for separating these monoallelic and biallelic *TP53*^*MT*^ (Additional file 1: Figures S8 & Fig. 3D). Accordingly, we classified the *TP53*^*MT*^ into three main groups: A) “obligatory” biallelic, B) “probable biallelic”, and C) “probable monoallelic” groups (Fig. 3E). Based on this approach, 579 (57%) patients had obligatory biallelic *TP53*^*MT*^, 239 (24%) had probable biallelic *TP53*^*MT*^, and 192 (19%) had probable monoallelic *TP53*^*MT*^ (Additional file 1: Table S5). The OS of patients with probable monoallelic *TP53*^*MT*^ (median OS: 29 [10–77]) was similar to the *TP53*^*WT*^ group (median: 42 [15–103]), *p* = 0.070. However, patients with probable biallelic *TP53*^*MT*^ (median OS: 14 [7–37]) had worse outcomes as compared to *TP53*^*WT*^* (p* < 0.001; Fig. 3C and Additional file 1: Fig. S9) at all VAF cutoffs. While the OS was similar when we compared AML to MDS within the obligatory biallelic *TP53*^*MT*^ group; however, AML showed a worse prognosis than comparable MDS, coinciding with higher *TP53*^*MT*^ VAFs in AML patients (Fig. 4A). Additionally, in comparison with monoallelic and probable biallelic groups, obligatory biallelic *TP53*^*MT*^ status was found to be associated more with pAML (OR: 1.84, [1.44–2.96]), sAML (OR: 2.50 [1.25–4.99]), complex karyotype (OR: 10.70 [5.20–21.9]), or carriers of del(17p) (OR: 5.42 [2.80–10.5]) (Fig. 4A, Additional file 1: Table S6 and S7).

To further highlight the limitations of the traditional classification methods, we applied our new algorithm to the previously classified single and double *TP53* hit groups. Indeed, we found significant OS differences between the new subgroups with the better prognostic resolution of the monoallelic/biallelic cases. Moreover, patients with probable biallelic *TP53* hits had survival rates between probable monoallelic *TP53*^*MT*^ and obligatory biallelic *TP53*^*MT*^ cases (Fig. 4B, Additional file 1: Fig. S10). This survival difference was also validated in an external confirmatory cohort (Additional file 1: Fig. S11).

### Confirming biallelic *TP53* inactivation and clonal mosaicism in selected cases

Our novel VAF-based method allowed a re-classification of 192 (19%) patients with single hits to probable biallelic class and 32 (3%) patients with double hits to probable monoallelic class of *TP53*^*MT*^ compared to the traditional classification (Additional file 1: Fig. S12). Accordingly, VAF < 50% in single or double *TP53* hits could be indeed associated with the presence of a subclone that acquired biallelic inactivation. To further confirm these results and characterize the subclonal configurations, we selected four patients (3 likely monoallelic and one likely biallelic *TP53*^*MT*^) and applied single-cell DNA mutational and copy number analysis. We found that some cells contained monoallelic *TP53*^*MT*^, while others had biallelic lesions in all cases studied. For example, in a pAML case (UPN13), the bulk NGS showed *TP53*^*MT*^ with a VAF of 8%; however, 6% of the cells were actually biallelic. In another HR-MDS case (UPN125), the bulk NGS showed *TP53*^*MT*^ with a VAF of 33%, while 4% were biallelic. In case (UPN423), NGS detected two *TP53*^*MT*^ with a combined VAF of 20%, but this sample contained 32% biallelic cells by single-cell DNA analysis. Finally, a case (UPN875) of LR-MDS had two *TP53*^*MT*^ (missense and truncated); however, the single-cell DNA analysis showed three *TP53*^*MT*^, and the percentage of biallelic cells was 34% (Additional file 1: Fig. S13A–D).

### Frequency of concurrent somatic mutations and factors associated with ***TP53***^***MT***^

We found that 41% of cases had *TP53*^*MT*^ as a sole molecular lesion, while the remaining 59% harbored additional somatic events. In particular, complex karyotype was more frequent among patients with isolated *TP53*^*MT*^ (*p* < 0.001). As to disease associations, LR-MDS cases had a lower burden of co-mutations, while the highest percentages were registered in MDS/MPN group (*p* = 0.0015; Additional file 1: Fig. S14). Interestingly, the rate of co-occurring events varied according to the *TP53*^*MT*^ status. The *TP53*^*WT*^ group had a rate of 2.10 co-mutation per patient. In contrast, this rate was lower in other groups with rates of 0.80, 1.42, and 1.76 co-mutations per patient in obligatory biallelic, probable biallelic, and probable monoallelic groups, respectively (Additional file 1: Fig. S15). We then applied our new classification scheme to investigate whether any difference was notable in the mutational configuration between patients with obligatory *vs.* probable monoallelic and probable biallelic *TP53*^*MT*^. In univariate analysis, we found that *IDH1, IDH2, EZH2, SUZ12, ASXL1, DNMT3A, JAK2, RUNX1, SF3B1, SRSF2, TET2*, and *U2AF1* mutations were less common in obligatory biallelic patients compared to probable monoallelic/probable biallelic cases; however, this correlation was not significant in a multivariate setting (Additional file 1: Table S8).

## Discussion

The prognostic impact of *TP53*^*MT*^ depends on the allelic configuration of the *TP53* hits reversely engineered a clinically applicable system enabling the imputation of allelic status that allows for a more precise and clinically applicable assignment of prognosis using routinely available molecular tools. The underlying hypothesis for our strategy was that traditionally defined single hit cases [11, 25] might also contain subclones with a biallelic *TP53* inactivation, which negatively influences prognosis.

Since in clinical situation the direct clonality measure is not available, we have proposed an algorithm that approximated allelic burden/copy number based on the cutoffs benchmarked according to the survival by applying a rationally developed strategy to our large and well-annotated cohort and provide a method for a more precise assessment of prognosis in carriers of *TP53* lesions under the assumption that the higher the clonal burden, the more likely is the presence of a cryptic biallelic subclone. Using a newly devised bioinformatics approach, we established a VAF cutoff for prognostic diversification of traditionally considered monoallelic *TP53*^*MT*^ groups. Conversely, we have also shown that double hits are not necessarily biallelic but may constitute a subclonal *TP53* mosaicism in a branching evolution mode. A more intricate method such as single-cell DNA sequencing, including the simultaneous analysis of mutations and CN-LOH, demonstrated the above-described points, in agreement with the observation in LFS. Indeed, using single-cell DNA sequencing we were able to discover the presence of biallelic clones carrying *TP53* lesions in cases with likely monoallelic hits, demonstrating that the analysis of clonal architecture at single-cell level is able to identify cryptic alterations not always detected by bulk sequencing. The critical size of the biallelic clone to affect the prognosis would be impossible to be precisely estimated, but as demonstrated by us a combined VAF may give away the presence and size of the double hit clones. Of note is that to date none of the studies systematically and directly addressed this issue in clinical setting due to obvious feasibility issues.

*TP53*^*MT*^ was common in MDS and AML patients in agreement with other studies [26, 27], while our earlier analyses have shown that a second *TP53* lesion was the most common hit associated with *TP53*^*MT*^ [14] and that second *TP53* hits are likely sweeping lesions [12]. Previous studies in MDS [11, 25] have already demonstrated that monoallelic lesions have no clinical impact on prognosis *vs*. *TP53*^*WT*^ cases, but biallelic clones defined by the presence of two *TP53* lesions have more aggressive phenotypes. We initially confirmed this finding according to traditional methods, but our current strategy, as described, further refined both double- and single-hit cases so that the latter could be sub-stratified according to the probability of the presence of truly biallelic subclones and thereby distinguished by worse outcomes and vice versa. For instance, patients with *TP53*^*MT*^, whether single or double hits and combined VAF of < 23% rarely harbor biallelic subclones and indeed show WT-like survival. Conversely, *TP53*^*MT*^ patients with a VAF of > 23% have more aggressive disease.

In our cohort, MDS/MPN has lower frequency of *TP53*^*MT*^ and lower clonal burdens and thus by inference the double hits should be less common. Indeed, other studies have shown that *TP53*^*MT*^ (independently from single or double hits) are relatively infrequent in MDS/MPN (< 5%) especially CMML accounting for 1–5% of the cases [28–30]. Although *TP53*^*MT*^ frequency increases in MDS/MPN-U [21] and therapy-related CMML (< 2%) [31], the presence of such lesions remains uncommon most likely due to the lower rate of transformation of patients with MDS/MPN compared to MDS. However, one can also stipulate that the initiation and progression of MN with myeloproliferative phenotype are more likely driven by RAS pathway alterations possibly mutually exclusive with *TP53*^*MT*^.

To highlight the limitations of previous traditional classifications of *TP53*^*MT*^, the International Consensus Classification of myeloid neoplasms and acute leukemias group recently published new classification schemes of MN with *TP53*^*MT*^. [32] Although the new classifications addressed the constraints of the previous schemes, the issue of possible subclonal mosaicism *vs*. truly biallelic hits and the possibility of cryptic biallelic hits in seemingly monoallelic cases remained unresolved. In our algorithm, we showed that patients with single *TP53*^*MT*^ and VAF more than 23% are acting biologically like biallelic *TP53*^*MT*^ cases. We think that *TP53* VAF is more accurate to determine the biallelic involvement than the presence of complex karyotypes unless the latter contain del(17p).

According to our proposed algorithm, MDS and AML with obligatory biallelic mutations had similar survival, thus upstaging MDS irrespective of the blast count. Recent findings that patients with AML have similar OS compared to those with MDS with excess blasts [25] overlapping with the presented results. Although probable biallelic group had worse outcome compared to probable monoallelic group, their OS was better compared to the obligatory biallelic *TP53*^*MT*^. This finding can be explained by the small biallelic clone among these patients contributing to a better OS when compared to the obligatory biallelic group.

Monoallelic *TP53*^*MT*^ are not unimportant as they constitute the first step in establishing a dominant clone characterized by biallelic *TP53* loss. Unexpectedly, we could not differentiate the prognosis between frameshift and dominant-negative missense mutations in any of the possible configurations.

Because our method relies on parameter optimization alone, while improving previous approaches, it has clear limitations. For instance, we did not consider the clonality of all samples since  these data were not readily available for all the cohorts in our analysis. However, such consideration will be necessary for future studies to estimate allelic involvement accurately.

In sum, our study demonstrates the importance of delineating the subclonal mosaicism of *TP53*^*MT*^ for modeling disease progression in MN. In addition to the genetic context, the role of *TP53*^*MT*^  may also vary in different disease subtypes (*e.g*., AML *vs.* MDS), and individual (single hit) VAF levels can shape patients' trajectories differently. In the future, our proposed approach could be incorporated into prognostication systems [16], such as IPSS-M to improve its precision with regard to patients’ outcomes. Ultimately, resolution of *TP53* inactivation status may proof a valuable tool for identifying the most suitable candidates for *TP53*-targeted therapeutic strategies.

### Supplementary Information

**Additional file 1**. Novel scheme for defining the clinical implications of TP53 mutations in myeloid neoplasia.

## Data Availability

All the data used to support our results are available in this article. NGS data of The Cleveland Clinic cohort can be requested by contacting the corresponding author (maciejj@ccf.org).
