# Supplementary material for: Novel scheme for defining the clinical implications of TP53 mutations in myeloid neoplasia
Source: J Hematol Oncol. 2023 Aug 3;16:91. doi: 10.1186/s13045-023-01480-y (PMC10401750; doi:10.1186/s13045-023-01480-y)
Supplement: Supplementary file 1 — Additional file 1. Novel scheme for defining the clinical implications of TP53 mutations in myeloid neoplasia. [file 13045_2023_1480_MOESM1_ESM.docx]

**SUPPLEMENTAL MATERIAL**

**Novel Scheme for Defining the Clinical Implications of *TP53* Mutations in Myeloid Neoplasia**

Waled Bahaj^1,2#^, Tariq Kewan^1,3#^, Carmelo Gurnari^1,4^, Arda Durmaz^1^, Ben Ponvilawan^1^, Ishani Pandit^1^, Yasuo Kubota^1^, Olisaemeka D. Ogbue^1^, Misam Zawit^1^, Yazan Madanat^5^, Taha Bat^5^, Suresh K. Balasubramanian^6^, Hussein Awada^1^, Ramsha Ahmed^1^, Minako Mori^1^, Manja Meggendorfer^7^, Torsten Haferlach^7^, Valeria Visconte^1*^, Jaroslaw P. Maciejewski^1*^

^1^Department of Translational Hematology and Oncology Research, Taussig Cancer Institute, Cleveland Clinic, Cleveland, OH.

^2^Division of Medical Oncology & Hematology, School of Medicine University of Louisville, Louisville, KY.

^3^Division of Hematology & Oncology, Yale School of Medicine, New Haven, CT.

^4^Department of Biomedicine and Prevention, PhD in Immunology, Molecular Medicine and Applied Biotechnology, University of Rome Tor Vergata, Rome, Italy.
^5^Department of Internal Medicine, Division of Hematology and Oncology, University of Texas Southwestern Medical Center, Dallas, TX.

^6^Department of Hematology and Oncology, Wayne State University, Detroit, MI.

^7^MLL Munich Leukemia Laboratory, Munich, Germany.

^#*^ These authors equally contributed to this work

***Corresponding authors:**

1) **Jaroslaw P. Maciejewski MD, Ph.D**

Department of Translational Hematology and Oncology Research, Taussig Cancer Institute

9620 Carnegie Ave N building, Building NE6-250, Cleveland, OH, USA 44106

E-mail: [maciejj@ccf.org](mailto:maciejj@ccf.org); ORCID ID: 0000-0002-6837-4346

2) **Valeria Visconte, Ph.D**

Department of Translational Hematology and Oncology Research, Taussig Cancer Institute

9620 Carnegie Ave n building, Building NE6-250, Cleveland, OH, USA 44106

E-mail: visconv@ccf.org; ORCID ID: 0000-0002-2993-1509

**Content:**

**A) Supplementary tables**

- **Supplementary Table-1.** Summary of the sources of myeloid neoplasms cases included.
- **Supplementary Table-2.** List of 46 genes included in our targeted sequencing panel.
- **Supplementary Table-3.** Univariate and multivariate logistic regression for *TP53* mutants *vs.* wild-type patients.
- **Supplementary Table-4**. Clinical and cytogenetics characteristics of the validation cohort.
- **Supplementary Table-5.** Characteristics of patients carrying *TP53* mutations (obligatory biallelic *vs.* others).
- **Supplementary Table-6.** Univariate and multivariate logistic regression for *TP53* mutations carriers (obligatory biallelic *vs.* probable monoallelic).
- **Supplementary Table-7.** Univariate and multivariate logistic regression for *TP53* mutations carriers (obligatory biallelic *vs.* probable biallelic).
- **Supplementary Table-8.** Univariate and multivariate logistic regression for *TP53* mutations carriers (probable biallelic *vs.* probable monollelic).
- **B) Supplementary figures**
- **Supplementary Figure 1.** Patients’ cohort disease subtypes.
- **Supplementary Figure 2.** Distribution of canonical missense mutations.
- **Supplementary Figure 3. C:** Number of patients with chromosome 17p deletion
- **Supplementary Figure 4.** Number of hits in *TP53* locus in each disease subtype.
- **Supplementary Figure 5.** Different configurations of *TP53* double hits mutations.
- **Supplementary Figure 6.** A new approach to assess *TP53* allelic inactivation.
- **Supplementary Figure 7**. Examples of *TP53* mutation with VAF of 20% with allelic inactivation probabilities in different settings.
- **Supplementary Figure 8.** Random forest survival analysis for determination of a VAF cutoff.
- **Supplementary Figure 9.** Kaplan-Meier survival estimates comparing groups according to new classification.
- **Supplementary Figure 10.** Kaplan-Meier survival estimates comparing single and double hits *TP53* groups in MDS and AML patients.
- **Supplementary Figure 11.** Kaplan-Meier survival estimates of the validation cohort.
- **Supplementary Figure-12** TP53 hits reclassification based on novel method
- **Supplementary Figure 13.** Single-cell DNA sequencing in selected samples.
- **Supplementary Figure 14.** Somatic co-mutations in different disease subtypes.
- **Supplementary Figure 15.** Frequency of concurrent somatic mutations.

**C) Supplementary figure legends, page 17**

**D) Supplementary Methods, page 18**

- Genetic studies
- Conventional cytogenetics
- Statistical Methods
- Single-cell DNA analysis

**E) References, page 29**

**Supplementary Table-1. Summary of the sources of myeloid neoplasm cases included in our study**

| Cohorts | Number of patients |
| --- | --- |
| Our cohorts | 3,319 |
| Cleveland Clinic Foundation (CCF) | 1,357 |
| Munich Leukemia Laboratory (MLL) | 1,962 |
| Public Cohorts | 4,081 |
| Memorial Sloan Kettering Cancer Center^1^ | 3,324 |
| Beat AML master trial^2^ | 436 |
| TCGA^3^ | 321 |
| External Validation Cohort |  |
| Wayne State University Karmanos Comprehensive Cancer Center | 53 |
| University of Texas Southwestern [Simmons Comprehensive Cancer Center](https://www.utsouthwestern.edu/departments/simmons/) | 39 |

1. Bernard E., et al. *Implications of TP53 allelic state for genome stability, clinical presentation and outcomes in myelodysplastic syndromes.* Nat Med. 2020;26(10):1549-1556.
2. Burd, A., et al., *Precision medicine treatment in acute myeloid leukemia using prospective genomic profiling: feasibility and preliminary efficacy of the Beat AML Master Trial.* Nat Med. 2020. 26(12): 1852-1858.
3. *The Cancer Genome Atlas Research Network. Genomic and epigenomic landscapes of adult de novo acute myeloid leukemia.* N Engl J Med. 2013;368:2059-2074.

**Supplementary Table-2. List of the 46 genes included in our targeted sequencing panel**

| *ABL1* | *ASXL1* | *BCOR* | *BCORL1* | *BRAF* |
| --- | --- | --- | --- | --- |
| *CALR* | *CBL* | *CEBPA* | *CHEK2* | *CSF3R* |
| *CUX1* | *DNMT3A* | *EP300* | *ETV6* | *EZH2* |
| *FLT3* | *GATA1* | *GATA2* | *IDH1* | *IDH2* |
| *JAK3* | *KDM6A* | *KIT* | *KMT2D* | *KRAS* |
| *JAK2* | *MPL* | *NF1* | *NOTCH1* | *NPM1* |
| *PHF6* | *PTPN11* | *RAD21* | *RUNX1* | *SETBP1* |
| *NRAS* | *SF3B1* | *SMC1A* | *SRSF2* | *STAG2* |
| *STAT3* | *SUZ12* | *TET2* | *U2AF1* | *WT1* |
| *ZRSR2* |  |  |  |  |

**Supplementary Table-3. Univariate and multivariate logistic regression for *TP53^MT^* *vs* *TP53^WT^***

| Variables | Univariate Analysis | | Multivariate Analysis | |
| --- | --- | --- | --- | --- |
|  | **Odds ratio (95% CI)** | **p-value** | **Odds ratio (95% CI)** | **p-value** |
| Gender (male *vs* female) | 0.808 (0.680-0.960) | 0.015 | 0.939 (0.71-1.75) | 0.508 |
| Disease subtypes |  |  |  |  |
| pAML | 1.340 (1.158-1.549) | <0.001 | 1.11 (0.61-2.04) | 0.706 |
| sAML | 4.929 (3.823-6.334) | <0.00 | 3.44 (1.74-6.77) | <0.001 |
| HR-MDS | 1.617 (1.376-1.901) | <0.001 | 0.74 (0.38-1.48) | 0.402 |
| LR-MDS | 0.579 (0.497-0.675) | <0.001 | 0.776 (0.41-1.46) | 0.431 |
| MDS/MPN | 0.672 (0.442-1.020) | 0.064 |  |  |
| MPN | 0.339 (0.197-0.583) | 0.000 | 0.766 (0.31-1.90) | 0.565 |
| CMML | 0.244 (0.163-0.366) | 0.000 | 0.537 (0.18-1.53) | 0.243 |
| Cytogenetic data |  |  |  |  |
| Normal | 0.108 (0.089-0.132) | 0.000 | 0.602 (0.38-0.95) | 0.029 |
| Complex | 18.80 (15.79-22.39) | 0.000 | 22.07 (14.77-32.98) | <0.001 |
| Deletion 5q | 3.430 (2.87-4.104) | 0.000 | 9.252 (5.61-15.27) | <0.001 |
| Deletion 7 | 0.540 (0.335-0.870) | 0.011 | 1.525 (0.79-2.94) | 0.208 |
| Deletion 17p | 4.870 (1.542-15.37) | 0.007 | 9.220 (2.17-39.06) | 0.003 |
| Deletion 20q | 0.320 (0.154-0.663) | 0.002 | 0.928 (0.34-2.52) | 0.883 |
| Trisomy 8 | 0.560 (0.391-0.803) | 0.002 | 1.312 (0.91-1.80) | 0.102 |
| Deletion Y | 0.462 (0.238-0.880) | 0.023 | 1.48 (0.55-3.98) | 0.438 |
| Next generation sequencing (mutant *vs* wild-type) |  |  |  |  |
| *RAS mutations* | 0.648 (0.518-0.811) | <0.001 | 0.816 (0.573-1.162) | 0.262 |
| *STAG2/SMC1A/RAD21* | 0.493 (0.314-0.775) | 0.002 | 0.869 (0.428-1.763) | 0.698 |
| *EZH2/SUZ12* | 0.753 (0.522-1.087) | 0.131 |  |  |
| *IDH1/IDH2* | 0.313 (0.220-0.444) | <0.001 | 0.687 (0.410-1.151) | 0.154 |
| *FLT3* | 0.118 (0.062-0.223) | <0.001 | 0.310 (0.142-0.675) | 0.003 |
| *ASXL1* | 0.308 (0.232-0.408) | <0.001 | 0.352 (0.213-0.582) | <0.001 |
| *DNMT3A* | 0.898 (0.715-1.128) | 0.358 |  |  |
| *JAK2* | 0.812 (0.572-1.154) | 0.247 |  |  |
| *NOTCH1* | 0.920 (0.423-2.001) | 0.835 |  |  |
| *NPM1* | 0.050 (0.022-0.114) | <0.001 | 0.210 (0.081-0.543) | 0.001 |
| *RUNX1* | 0.333 (0.234-0.473) | <0.001 | 0.550 (0.300-1.007) | 0.053 |
| *SF3B1* | 0.604 (0.456-0.784) | <0.001 | 0.715 (0.446-1.146) | 0.164 |
| *SRSF2* | 0.251 (0.174-0.363) | <0.001 | 0.484 (0.257-0.911) | 0.025 |
| *TET2* | 0.660 (0.531-0.819) | <0.001 | 0.975 (0.659-1.441) | 0.900 |
| *U2AF1* | 0.843 (0.600-1.184) | 0.326 | 1.091 (0.616-1.932) | 0.763 |

CI: confidence interval, pAML: primary acute myeloid leukemia, sAML: secondary acute myeloid leukemia, HR-MDS: high-risk myelodysplastic syndrome, LR-MDS: low-risk myelodysplastic syndrome, MDS/MPN: myelodysplastic /myeloproliferative overlap syndrome, MPN, myeloproliferative syndrome, CMML: chronic myelomonocytic leukemia.

**Supplementary Table-4. Clinical and cytogenetic characteristics of the validation cohort**

| Characteristics | Patients |
| --- | --- |
| Number of patients | 92 |
| Median age at diagnosis (IQR) | 70.3 (61-77) |
| Male gender % | (58%) |
| Disease subtypes |  |
| pAML | 2 |
| sAML | 26 |
| HR-MDS | 28 |
| LR-MDS | 35 |
| MDS/MPN | 1 |
| Cytogenetic data |  |
| Normal | 20 |
| Complex | 72 |
| Deletion 5q | 40 |
| Deletion 7 | 12 |
| Deletion 17p | 16 |
| Deletion 20q | 7 |
| Trisomy 8 | 16 |

**Supplementary Table-5. Characteristics of patients (obligatory biallelic *TP53^MT^* *vs* others)**

| Characteristics | Probable mono/biallelic  N (%) | Obligatory  Biallelic  N (%) | p-value |
| --- | --- | --- | --- |
| Number of patients | 431 (42.7) | 579 (57.3) |  |
| Median age at diagnosis (IQR) | 71 (64-78) | 71 (63-76) | 0.64 |
| Male gender % | 55% | 53% | 0.95 |
| Disease subtype |  |  |  |
| pAML | 88 (20.9%) | 228 (38.6%) | <0.001 |
| sAML | 23 (5.4%) | 88 (14.9%) | <0.001 |
| HR-MDS | 102 (24.2%) | 132 (22.3%) | 0.36 |
| LR-MDS | 152 (36.1%) | 91 (15.4%) | <0.001 |
| MDS/MPN | 16 (3.8%) | 10 (1.6%) | 0.04 |
| MPN | 7 (2.1%) | 7 (1%) | 0.12 |
| CMML | 15 (3.5%) | 10 (1.6%) | 0.06 |
| Cytogenetic data |  |  |  |
| Normal | 105 (25%) | 20 (3.3%) | <0.001 |
| Complex | 178 (42.3%) | 526 (89.1%) | <0.001 |
| Deletion 5q | 75 (17.8%) | 20 (3.3%) | <0.001 |
| Deletion 7 | 14 (3.3%) | 6 (1%) | 0.01 |
| Deletion 17p | 12 (2.8%) | 153 (25.9%) | <0.001 |
| Deletion 20q | 7 (1.6%) | 1 (0.1%) | 0.02 |
| Trisomy 8 | 27 (6.4%) | 10 (1.6%) | <0.001 |
| Deletion Y | 9 (2.1%) | 1 (0.1%) | 0.006 |
| Median Hb (IQR), g/dL | 9.4 (8.3-10.7) | 9.0 (8.1-9.9) | 0.003 |
| Median WBC (IQR), x10^9^ /L | 4.3 (2.6-7.4) | 5.9 (2.6-20.3) | 0.002 |
| Median platelet (IQR), x10^9^ /L | 94 (48-204) | 52 (28-94) | <0.001 |
| Survival status, dead (%) | 211 (50.23%) | 383 (64.9%) | <0.001 |

N: number of patients, IQR: interquartile range, Hb: hemoglobin, WBC: white blood cells. pAML: primary acute myeloid leukemia, sAML: secondary acute myeloid leukemia, HR-MDS: high-risk myelodysplastic syndrome, LR-MDS: low-risk myelodysplastic syndrome, MDS/MPN: myelodysplastic /myeloproliferative overlap syndrome, MPN, myeloproliferative syndrome, CMML: chronic myelomonocytic leukemia.

**Supplementary Table-6. Univariate and multivariate logistic regression for Obligatory biallelic *TP53^MT^* (obligatory biallelic vs probable monoallelic)**

| Variables | Univariate Analysis | | Multivariate Analysis | |
| --- | --- | --- | --- | --- |
|  | **Odds ratio (95% CI)** | **P value** | **Odds ratio (95% CI)** | **P value** |
| Gender (male *vs* female) | .8823529 (0.5369455 -1.449955) | 0.621 |  |  |
| Disease subtype |  |  |  |  |
| pAML | 3.660397(2.378397 -5.63342) | <0.001 | 2.525666(1.214366 - 5.252934) | 0.013 |
| sAML | 11.19177(3.495742-35.83091) | <0.001 | 7.378835 (1.852713 -29.38783) | 0.005 |
| HR-MDS | .9805546( .658803-1.459446) | 0.923 |  |  |
| LR-MDS | .2 (.1386932 -.2884064) | <0.001 | .7329967(.4069109- 1.320397) | 0.301 |
| MDS/MPN | .4029304 (.1565971 - 1.036755) | 0.059 |  |  |
| MPN | .4564663 (0.1431002-1.456053) | 0.185 |  |  |
| CMML | .5433455 ( .1947243 - 1.516115) | 0.244 |  |  |
| Cytogenetic data |  |  |  |  |
| Normal | .056719 (.0332226 -.0968329) | <0.001 | 1.101543 (.378527 -3.205578) | 0.859 |
| Complex | 31.07365(19.92199-48.46763) | <0.001 | 22.58034(8.976937 -56.79799) | <0.001 |
| Deletion 5q | .1478743(.0833308 - .2624096) | <0.001 | .9174619 (.310739 - 2.708821) | 0.876 |
| Deletion 7 | .3891892( .1173781 - 1.29043) | 0.123 |  |  |
| Deletion 17p | 8.617797(3.955061 -18.77757) | <0.001 | 1.855474 (.7714137-4.462954) | 0.167 |
| Deletion 20q | .1083334 ( .0111995 -1.047912) | 0.055 |  |  |
| Trisomy 8 | .3549103 (.1419498 -.8873648) | 0.027 | 2.263205(.4715648-10.86192) | 0.307 |
| Deletion Y | .0454082 (.005549 -.3715775) | 0.004 | .0952977 ( .0071262 - 1.274406) | 0.076 |
| Next generation sequencing (mutant *vs* wild-type) |  |  |  |  |
| *NF1/PTPN11/CBL* | .8109453 (.4600922 -1.429349) | 0.469 |  |  |
| *RAS mutations* | 1.110047 (.5056921 -2.436668) | 0.795 |  |  |
| *EZH2/SUZ12* | .4415313 (.1841774 -1.05849) | 0.067 |  |  |
| *IDH1/IDH2* | .5858586 (.2459472-1.395545) | 0.227 |  |  |
| *FLT3* | .5479452( .1214137-2.4729) | 0.434 |  |  |
| *ASXL1* | .3452632 (.1751465- .6806113) | 0.002 | .7316902(.2528508 2.117338) | 0.564 |
| *DNMT3A* | .5780856 (.3512601 -.9513832) | 0.031 | .8749568 ( .4132914 -1.852323) | 0.727 |
| *JAK2* | .4363636 (.1889438 - 1.007777) | 0.052 |  |  |
| *NOTCH1* | 1.6621 (.1845138-14.9722) | 0.651 |  |  |
| *NPM1* | 1.243736 (.1285241 -12.03571) | 0.851 |  |  |
| *RUNX1* | .3080876 (.1371266 -.6921921) | 0.004 | 1.104757 (.3087553- 3.952926) | 0.878 |
| *SF3B1* | .1207764 (.0637372 -.2288606) | <0.001 | .4033817( .1517382 - 1.072353) | 0.069 |
| *SRSF2* | .3298612(.1399082- .7777128) | 0.011 | .5025355 (.1170349- 2.157835) | 0.355 |
| *TET2* | .3340441 (.2080274 -.2080274 ) | <0.001 | .7941261 (.3684426-1.711627) | 0.556 |
| *U2AF1* | .5352113 ( .2480039 - 1.155027) | 0.111 |  |  |

CI: confidence interval, pAML: primary acute myeloid leukemia, sAML: secondary acute myeloid leukemia, HR-MDS: high-risk myelodysplastic syndrome, LR-MDS: low-risk myelodysplastic syndrome, MDS/MPN: myelodysplastic /myeloproliferative overlap syndrome, MPN, myeloproliferative syndrome, CMML: chronic myelomonocytic leukemia.

**Supplementary Table-7. Univariate and multivariate logistic regression for Obligatory biallelic *TP53^MT^* (obligatory biallelic vs probable biallelic)**

| Variables | Univariate Analysis | | Multivariate Analysis | |
| --- | --- | --- | --- | --- |
|  | **Odds ratio (95% CI)** | **P value** | **Odds ratio (95% CI)** | **P value** |
| Gender (male *vs* female) | .9808434 (.6718812 -1.431881) | 0.920 |  |  |
| Disease subtype |  |  |  |  |
| pAML | 1.908594 ( 1.361269-2.675982) | <0.001 | 1.202536 (.5188436 - 2.787145 ) | 0.667 |
| sAML | 1.855011 (1.121131 - 3.069279) | 0.016 | 1.285027 (.4872002 - 3.389354) | 0.612 |
| HR-MDS | .6952027 ( .4918732- .982584) | 0.039 | .5898481 (.2638343 -1.318709 ) | 0.198 |
| LR-MDS | .5334374 (.3693314 - .770461) | 0.001 | .6989374 ( .3054567 - 1.599289) | 0.396 |
| MDS/MPN | .5105311(.1988998 - 1.310419) | 0.162 |  |  |
| MPN | 1.459927(.3010056 -7.080888) | 0.639 |  |  |
| CMML | .4517705 ( .1811307 - 1.126791) | 0.088 |  |  |
| Cytogenetic data |  |  |  |  |
| Normal | .2345194 ( .1311102 -.4194896) | <0.0001 | 1.763227 (.5876062 -5.290907) | 0.312 |
| Complex | 7.59705 (5.099829 -11.31708) | <0.0001 | 7.936569 (3.192213 - 19.73212) | <0.0001 |
| Deletion 5q | .1916529 (.1088759-.3373642 ) | <0.0001 | 1.084379 ( .3865264-3.042165) | 0.878 |
| Deletion 7 | .2714721 ( .0955218 - .7715212) | 0.014 | .805126 ( .1532554- 4.229723) | 0.798 |
| Deletion 17p | 4.638723 (2.697586 -7.97667) | <0.0001 | 2.499633 (1.360159-4.593701) | 0.003 |
| Deletion 20q | .1031278 (.0114653 -.9276092) | 0.043 | .6614969 ( .0272072-16.08319 ) | 0.800 |
| Trisomy 8 | .2187941 (.099416 -.4815209) | <0.0001 | 1.104781 (.3103623 -3.932635) | 0.878 |
| Deletion Y | .2080392 ( .018773 - 2.305456) | 0.201 |  |  |
| Next generation sequencing (mutant *vs* wild-type) |  |  |  |  |
| *NF1/PTPN11/CBL* | 1.608624 (.8252323 -3.135687) | 0.163 |  |  |
| *RAS mutations* | 1.632262 ( .6916226 -3.852217) | 0.263 |  |  |
| *EZH2/SUZ12* | .3249807 (.1465413 -.7207009) | 0.006 | .6445766 ( .2425529 - 1.712942) | 0.378 |
| *IDH1/IDH2* | .4155844 ( .191681 -.9010304) | 0.026 | .601833 (.2258616 -1.60365 ) | 0.310 |
| *FLT3* | .6179604 ( .1370376 - 2.786644) | 0.531 |  |  |
| *ASXL1* | .3345455 ( .1735821 -.6447708) | 0.001 | .6708306 (.2924627 -1.538704) | 0.346 |
| *DNMT3A* | .6649874 ( .4054012- 1.090792) | 0.106 |  |  |
| *JAK2* | .31026 (.1453135-.6624387) | 0.002 | .4662726 .1686445 -1.289162 ) | 0.141 |
| *NOTCH1* | .6179604 ( .1370376-2.786644) | 0.531 |  |  |
| *NPM1* | .6970387 (.1155792 -4.203725) | 0.694 |  |  |
| *RUNX1* | .4524362 (.1928608 - 1.061379) | 0.068 |  |  |
| *SF3B1* | .3042057 (.1504103-.6152579) | 0.001 | .7839101 (.322549 - 1.905184) | 0.591 |
| *SRSF2* | .4537037 (.1858272 - 1.107734) | 0.083 |  |  |
| *TET2* | .5410448 ( .3289126-.8899916) | 0.016 | 1.022831 (.5330182 -1.962755) | 0.946 |
| *U2AF1* | .4782473 ( .2316759 - .9872431) | 0.046 | .8749417 (.1516547-1.449088) | 0.188 |

CI: confidence interval, pAML: primary acute myeloid leukemia, sAML: secondary acute myeloid leukemia, HR-MDS: high-risk myelodysplastic syndrome, LR-MDS: low-risk myelodysplastic syndrome, MDS/MPN: myelodysplastic /myeloproliferative overlap syndrome, MPN, myeloproliferative syndrome, CMML: chronic myelomonocytic leukemia.

**Supplementary Table-8. Univariate and multivariate logistic regression for probable biallelic *TP53^MT^* (probable biallelic vs probable monoallelic)**

| Variables | Univariate Analysis | | Multivariate Analysis | |
| --- | --- | --- | --- | --- |
|  | **Odds ratio (95% CI)** | **P value** | **Odds ratio (95% CI)** | **P value** |
| Gender (male *vs* female) | .899586 (.5145087 -1.572869) | 0.710 |  |  |
| Disease subtype |  |  |  |  |
| pAML | 1.91785 (1.171679 -3.139211) | 0.010 | 1.162644 (.5964024-2.266493) | 0.658 |
| sAML | 6.03333 (1.770672-20.55777) | <0.0001 | 3.774252 (.9854065-14.45594) | 0.053 |
| HR-MDS | 1.410459 (.903298-2.202367) | 0.130 |  |  |
| LR-MDS | .3749268 (.2487871-.5650218) | <0.0001 | .5817977 (.3508296 -.9648233) | 0.036 |
| MDS/MPN | .7892377 (.2904289 -2.144746) | 0.643 |  |  |
| MPN | .3126638 (.0599606-1.630381) | 0.168 |  |  |
| CMML | 1.202702 (.4201792-3.442561) | 0.731 |  |  |
| Cytogenetic data |  |  |  |  |
| Normal | .2418517 (.1503619-.3890096) | <0.0001 | .4556837 (.2527777-.8214634) | 0.009 |
| Complex | 4.090226 (2.693512-6.211201) | <0.0001 | 2.269801 (1.332083-3.867622) | 0.003 |
| Deletion 5q | .7715736 (.4678101-1.27258) | 0.310 |  |  |
| Deletion 7 | 1.433628 (.4721759-4.352806) | 0.525 |  |  |
| Deletion 17p | 1.857795 (.7478515-4.615091) | 0.182 |  |  |
| Deletion 20q | 1.050505 (.2321833-4.752972) | 0.949 |  |  |
| Trisomy 8 | 1.62212 (.7112373-3.699571) | 0.250 |  |  |
| Deletion Y | 1.050505 (.2321833-4.752972) | 0.949 |  |  |
| Next generation sequencing (mutant *vs* wild-type) |  |  |  |  |
| *NF1/PTPN11/CBL* | .5041237 (.2392384 -1.062291) | 0.072 |  |  |
| *RAS mutations* | .680067 (.2480753-1.864318) | 0.454 |  |  |
| *EZH2/SUZ12* | 1.358639 (.5947055-3.103888) | 0.467 |  |  |
| *IDH1/IDH2* | 1.409722 (.5952595-3.338572) | 0.435 |  |  |
| *FLT3* | .8866995 (.1767384-4.448585) | 0.884 |  |  |
| *ASXL1* | 1.032037 (.5393535-1.974771) | 0.924 |  |  |
| *DNMT3A* | .8693182 (.5013109-1.507476) | 0.618 |  |  |
| *JAK2* | 1.406445 (.6408439-3.086694) | 0.395 |  |  |
| *NOTCH1* | 2.689655 (.2773195-26.08632) | 0.393 |  |  |
| *NPM1* | 1.784314 (.1604575 -19.84186) | 0.638 |  |  |
| *RUNX1* | .6809524 (.3010804-1.540107) | 0.356 |  |  |
| *SF3B1* | .3970223 (.2220049-.7100147) | 0.002 | .7668329 (.3951949-1.487956) | 0.432 |
| *SRSF2* | .7270408 (.3064571-1.724836) | 0.470 |  |  |
| *TET2* | .6174056 (.370484 -1.028896) | 0.064 |  |  |
| *U2AF1* | 1.11911 (.5096138-2.457561) | 0.779 |  |  |

CI: confidence interval, pAML: primary acute myeloid leukemia, sAML: secondary acute myeloid leukemia, HR-MDS: high-risk myelodysplastic syndrome, LR-MDS: low-risk myelodysplastic syndrome, MDS/MPN: myelodysplastic /myeloproliferative overlap syndrome, MPN, myeloproliferative syndrome, CMML: chronic myelomonocytic leukemia.

**Supplementary Figure-1** Patients’ cohort disease subtypes.


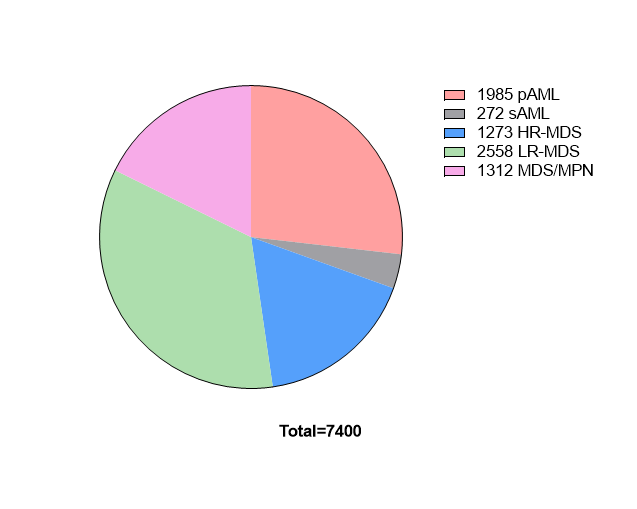


**Supplementary Figure-2** Distribution of canonical missense mutations.

**Supplementary Figure-3. C: Number of patients with chromosome 17p deletion**

**
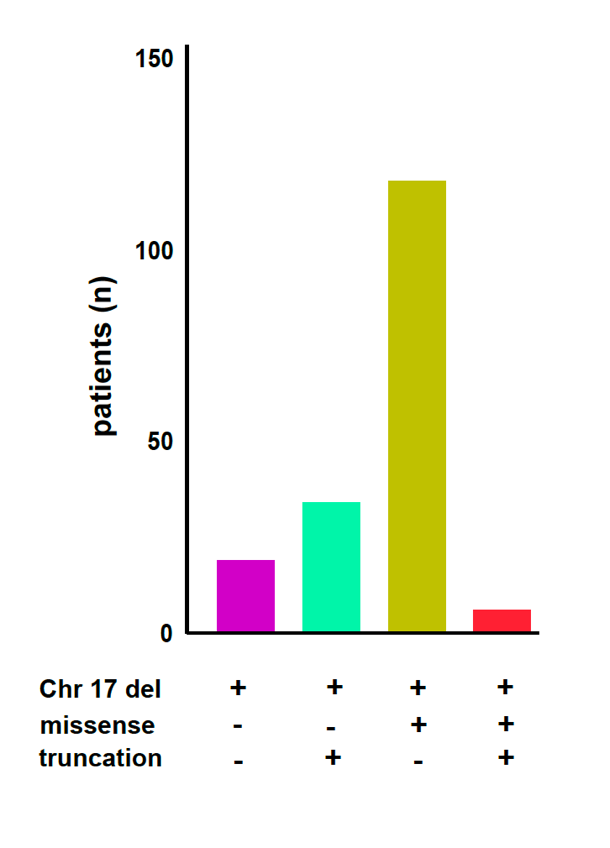
**

**Supplementary Figure-4** Number of hits in *TP53* locus in each disease subtype.

**
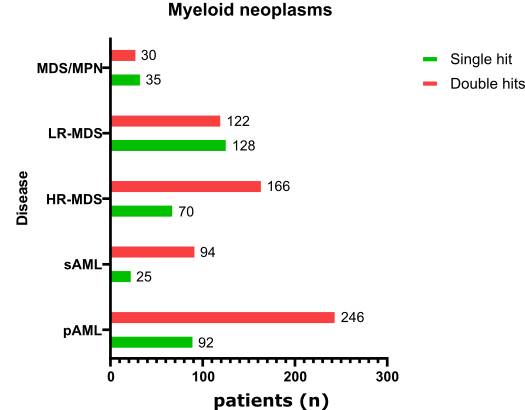
**

- **Supplementary Figure-5** Different configurations of *TP53* double hits mutations.

**Supplementary Figure-6** A new approach to assess *TP53* allelic inactivation.


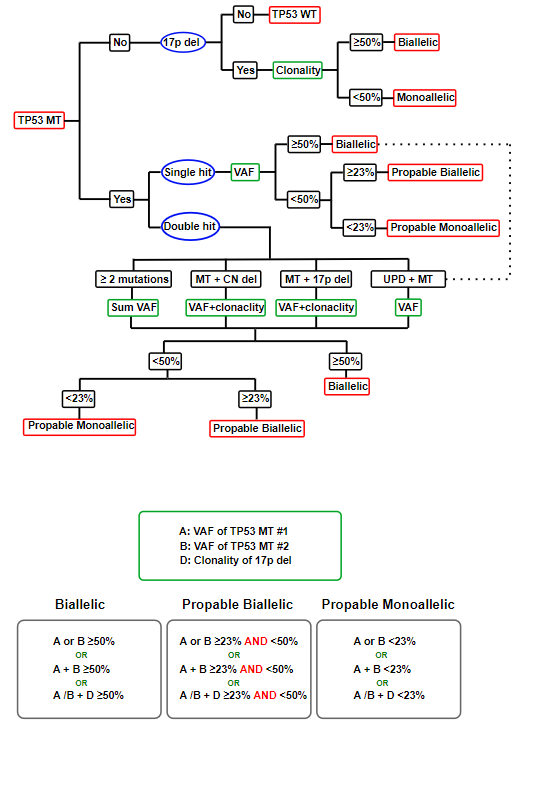


**Supplementary Figure-7** Examples of *TP53* mutation with VAF of 20% with allelic inactivation probabilities in different settings.

**
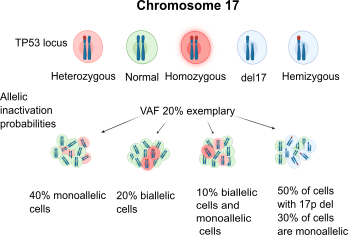
**

**Supplementary Figure-8** Random forest survival analysis for determination of a VAF cutoff.


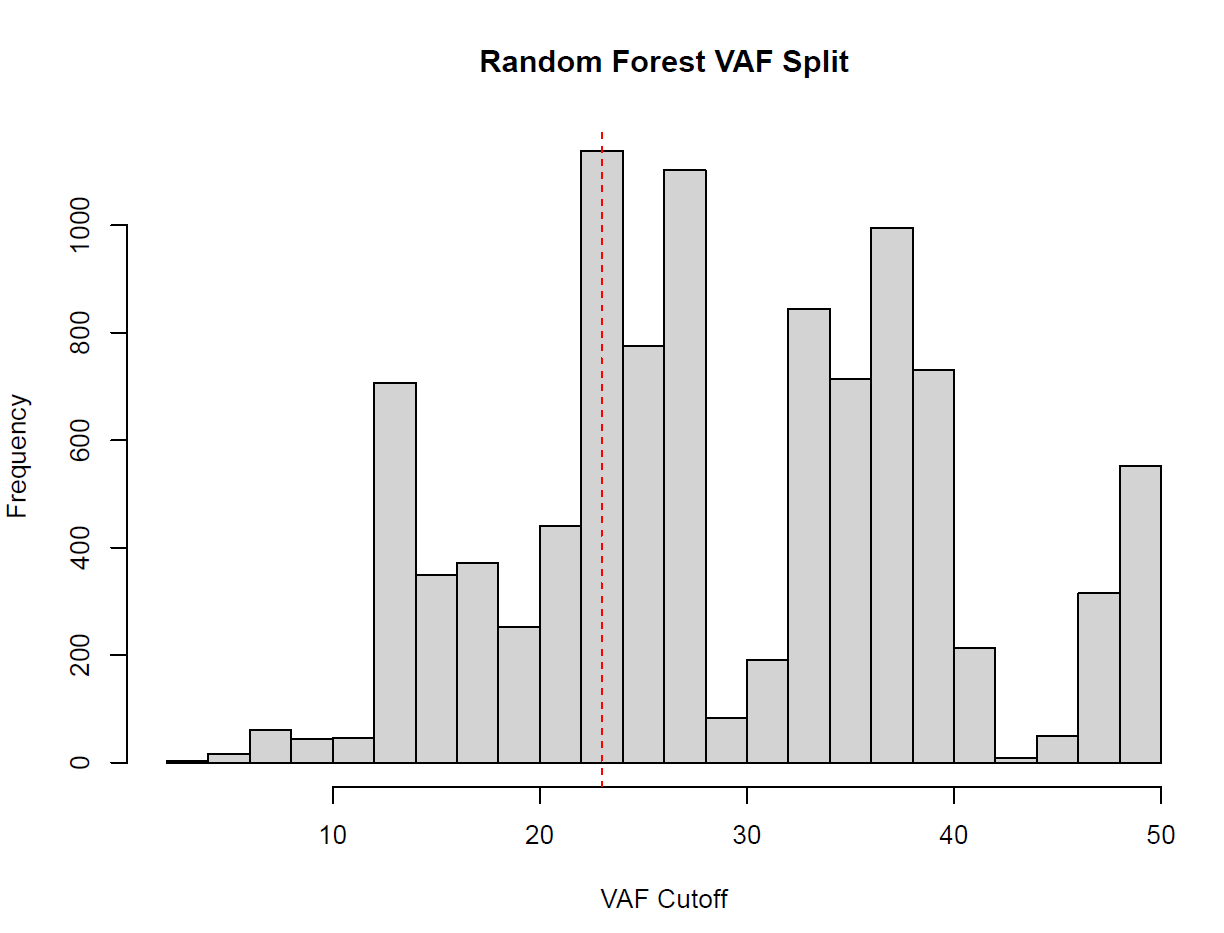


**Supplementary Figure 9** Kaplan-Meier survival estimates comparing groups according to new classification.


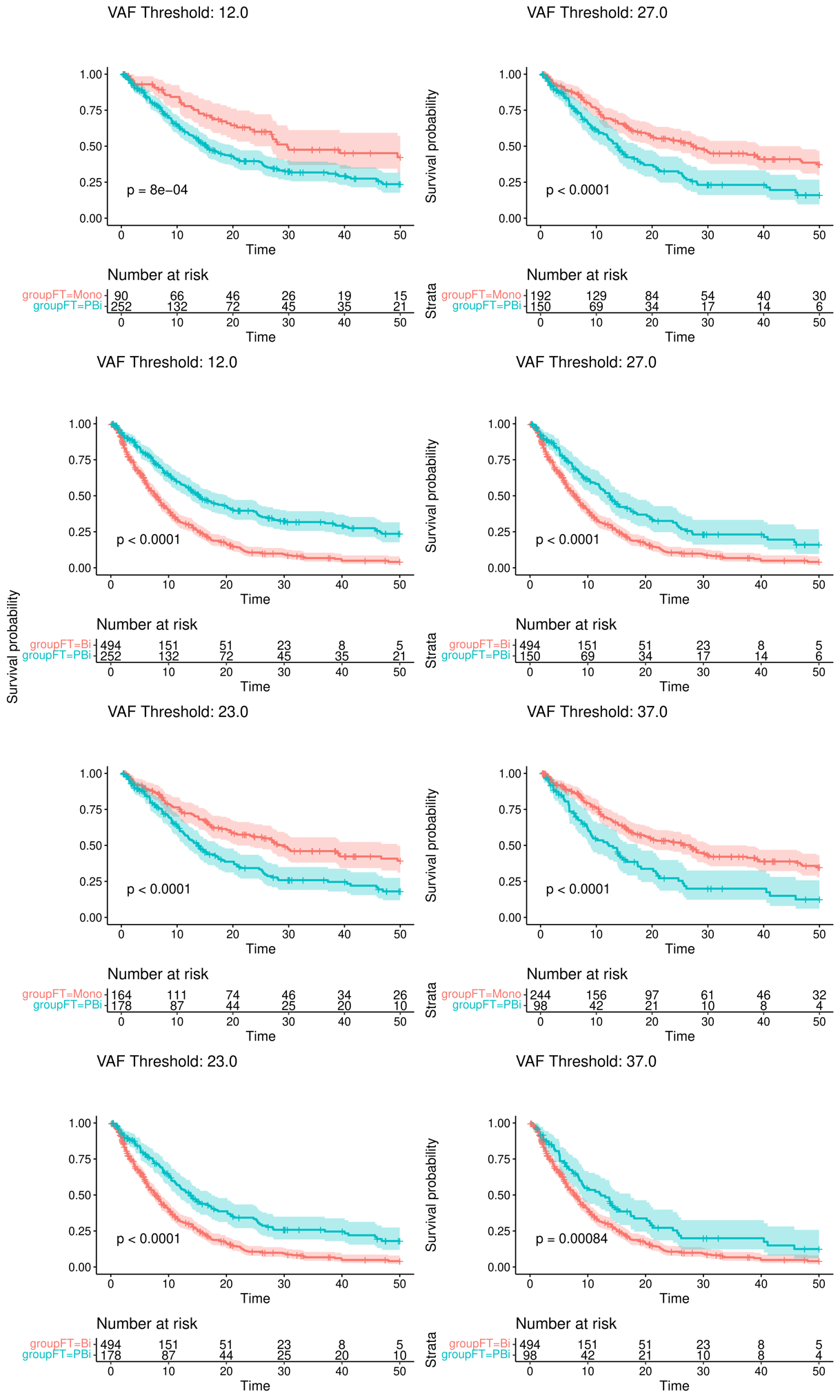


**Supplementary Figure-10** Kaplan-Meier survival estimates comparing single and double hits *TP53* groups in MDS and AML patients.

**
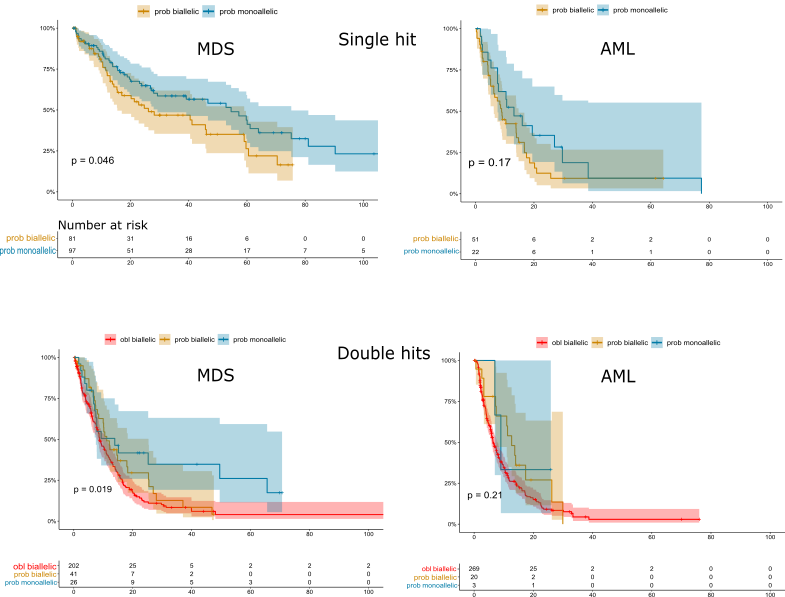
**

**Supplementary Figure-11** Kaplan-Meier survival estimates of the validation cohort.

**
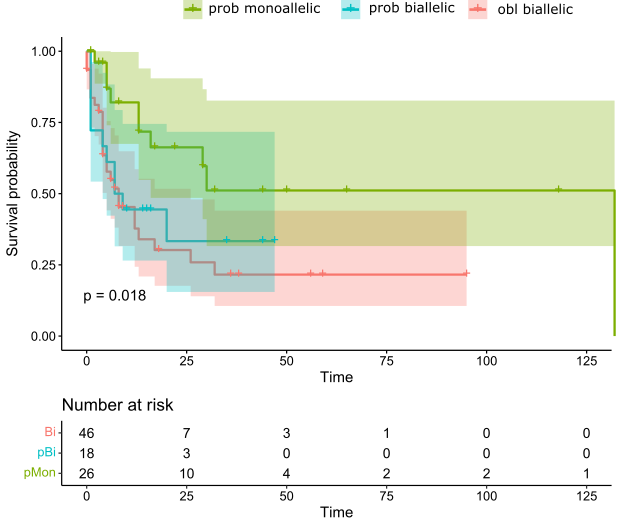
**

**Supplementary Figure-12** TP53 hits reclassification based on novel method

**
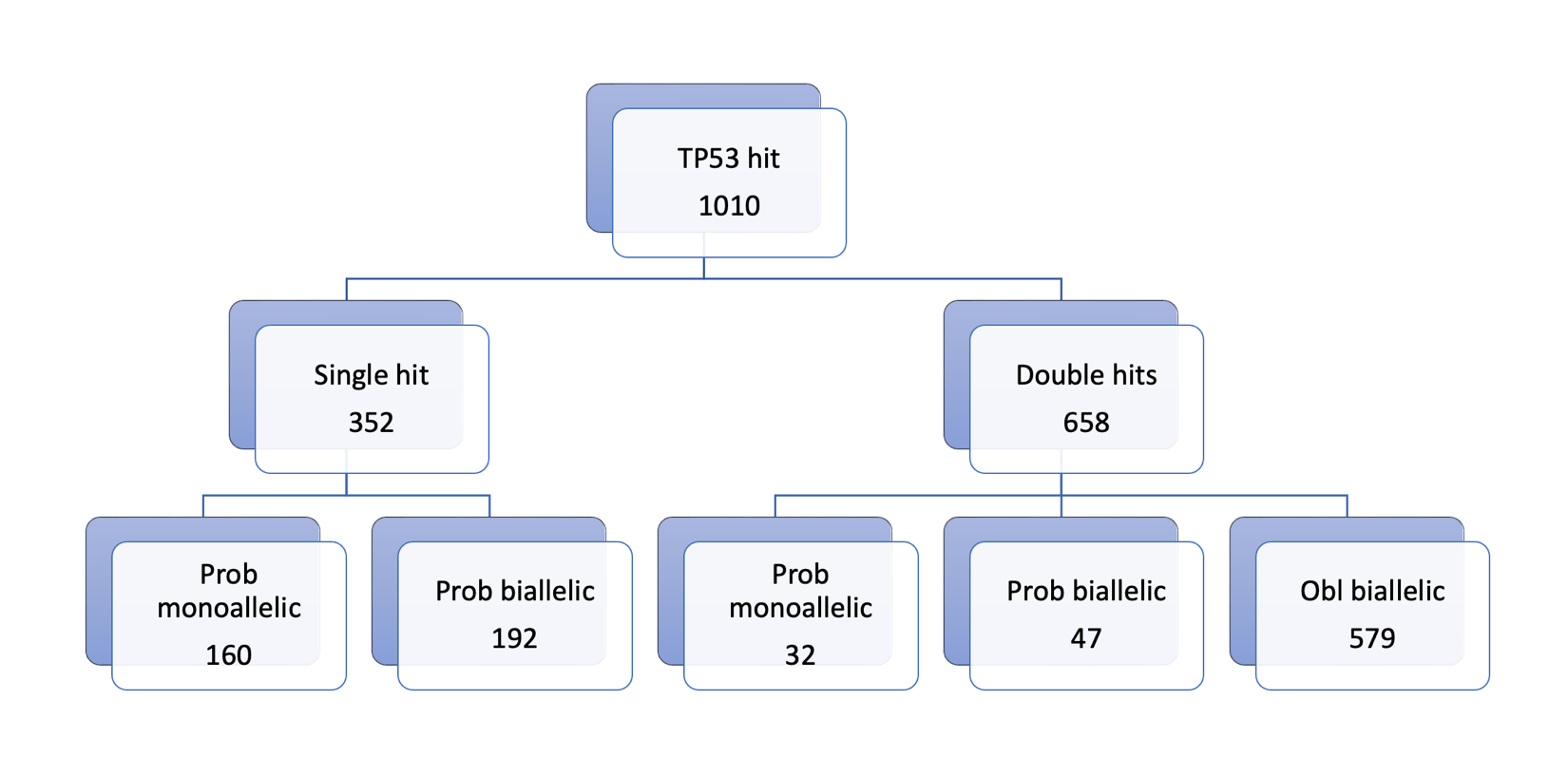
**

**Supplementary Figure-13** Single-cell DNA sequencing in selected samples.

**A**


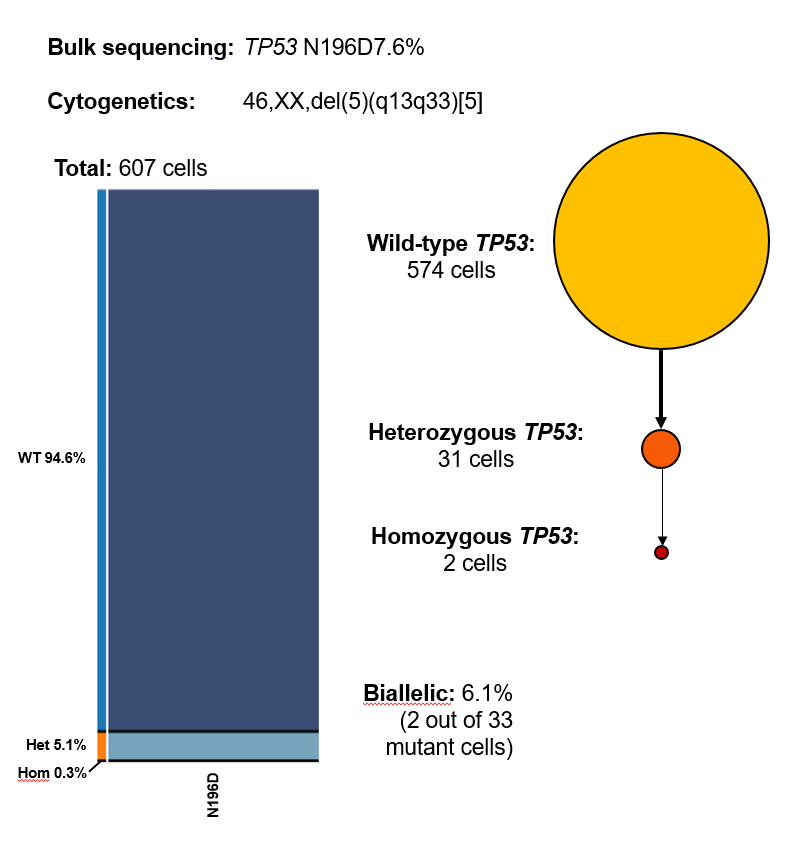


**B**


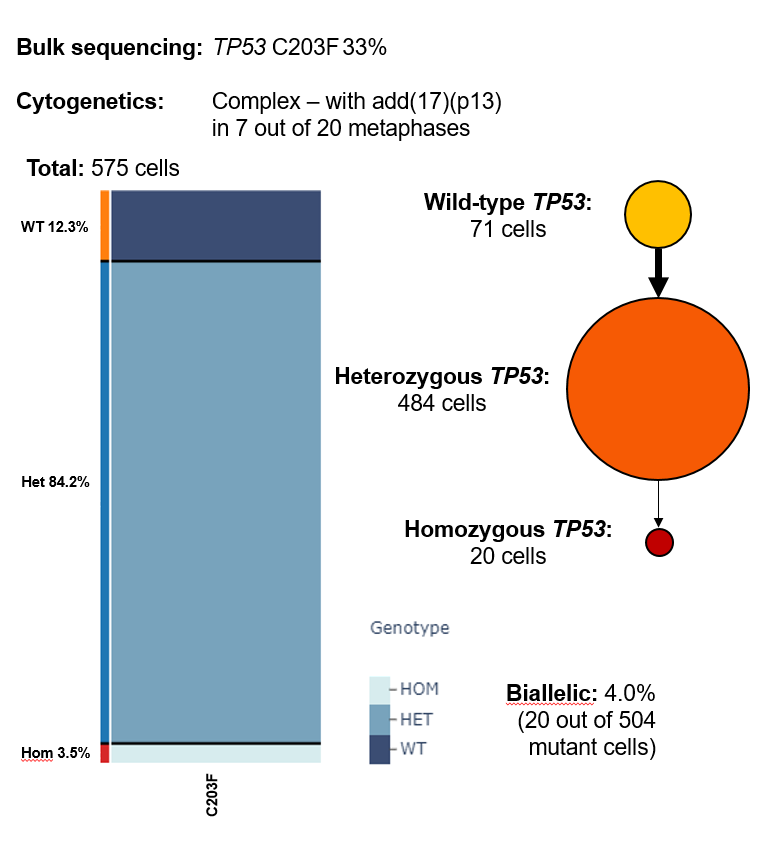


**C**

**
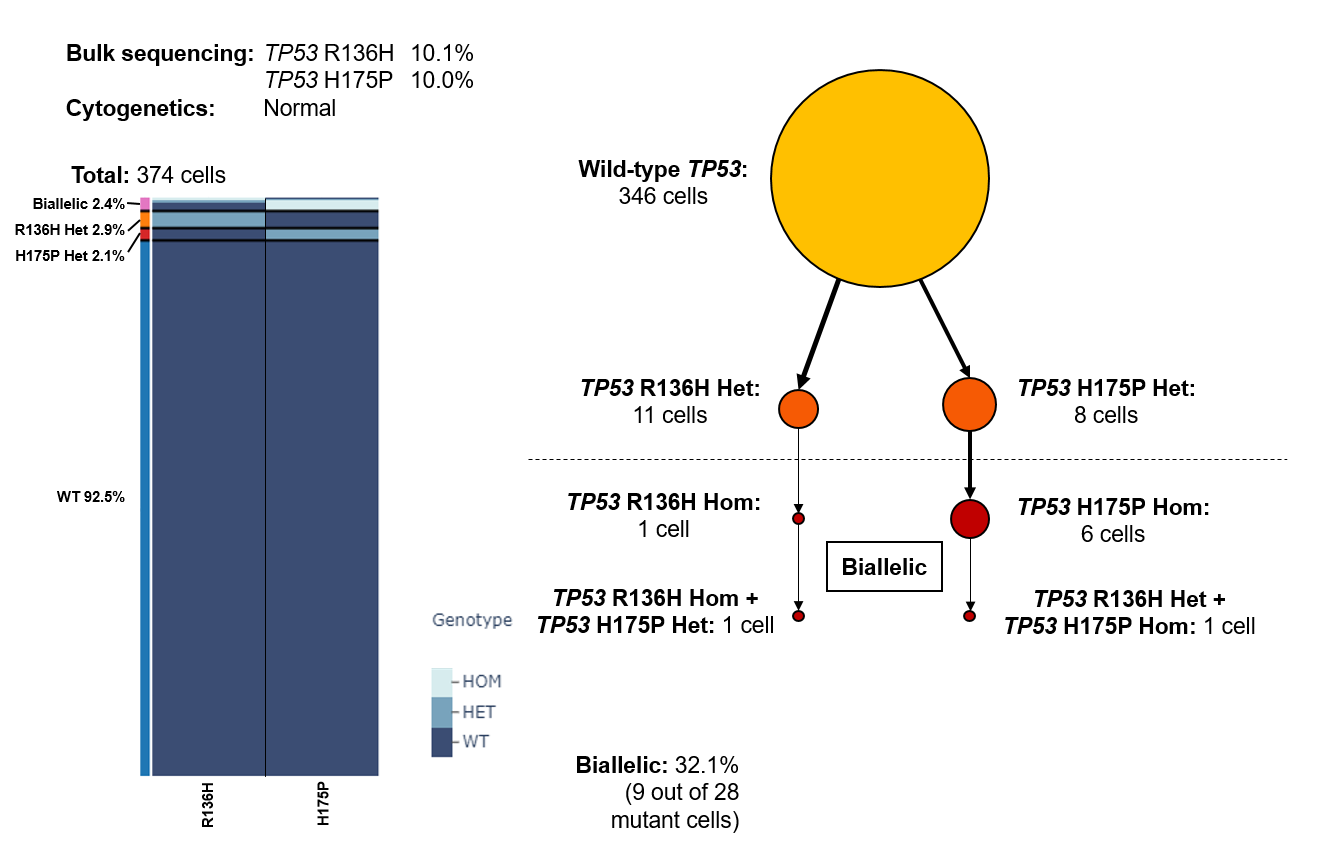
**

**D**

**
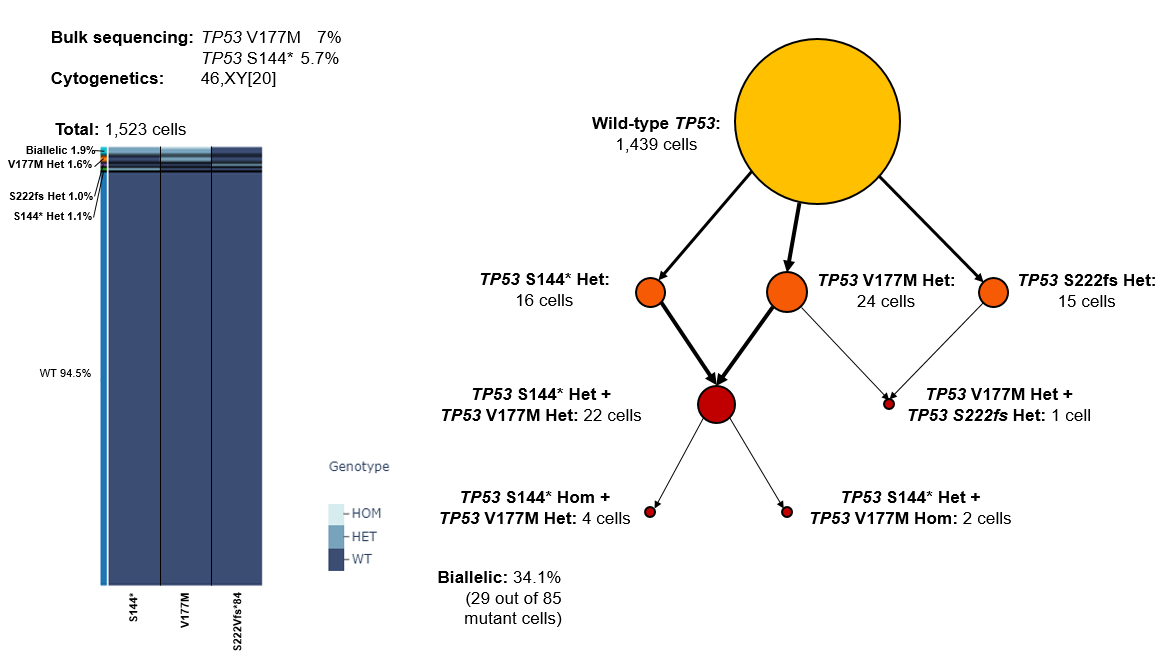
**

**Supplementary Figure-14** Somatic co-mutations in different disease subtypes.

**Supplementary Figure-15** Frequency of concurrent somatic mutations.


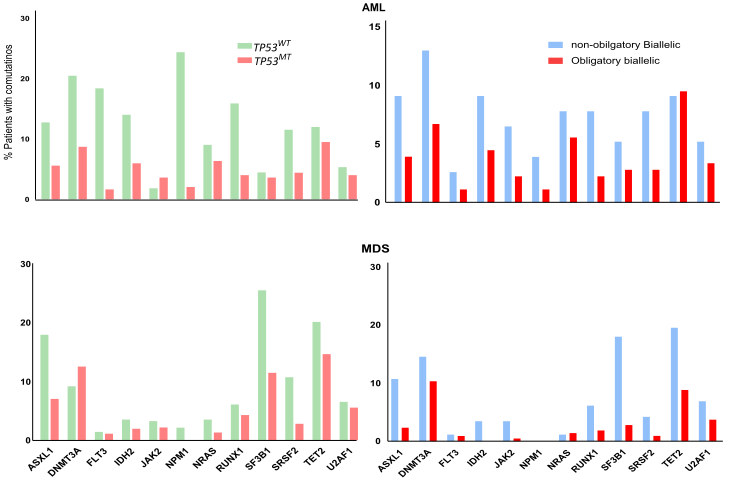


**Supplementary figure legends**

**Supplementary Figure 1. Patients’ cohort disease subtypes.** Pie chart presenting the total number of patients within each disease subtype included in our study. pAML: primary acute myeloid leukemia, sAML: secondary acute myeloid leukemia, HR-MDS: high-risk myelodysplastic syndrome, LR-MDS: low-risk myelodysplastic syndrome, MDS/MPN: myelodysplastic/myeloproliferative overlap syndrome.

**Supplementary Figure 2. Distribution of canonical missense mutations.** Number of patients with hotspot-canonical missense *TP53^MT^* sites. R1-R6 indicate the canonical hotspots (*see Figure 1 in the text*). *TP53^MT^*: *TP53* mutation.

**Supplementary Figure 3. C: Number of patients with chromosome 17p deletion**. **Number of patients with chromosome 17p deletion, including those with deletion only, deletion with truncated mutation, and** **deletion with missense mutations.**

**Supplementary Figure 4. Number of hits in *TP53* locus in each disease subtype.** The frequency of single and double *TP53* hits within each disease subtype. pAML: primary acute myeloid leukemia, sAML: secondary acute myeloid leukemia, HR-MDS: high-risk myelodysplastic syndrome, LR-MDS: low-risk myelodysplastic syndrome, MDS/MPN: myelodysplastic/myeloproliferative overlap syndrome.

**Supplementary Figure 5.** **Different configurations of *TP53* double hits mutations.** Different canonical sites in *TP53* double hits configurations. Ms: missense, trunc: truncation.

**Supplementary Figure 6. A new approach to assess *TP53* allelic inactivation.** A novel flow algorithm for the precise classification of *TP53^MT^* into obligatory biallelic, probable biallelic, or probable monoallelic groups. *TP53^MT^*: *TP53* mutation, *TP53^WT^*: *TP53* wild-type, VAF: variant allele frequency, CN del: copy number deletion, UPD: uniparental disomy.

**Supplementary Figure 7. Examples of *TP53* mutation with VAF of 20% with allelic inactivation**. Examples of *TP53* mutation with variant allele frequency (VAF) of 20% showing different probabilities of allelic inactivation in different configurations.

**Supplementary Figure 8.** **Random forest survival analysis for determination of a VAF cutoff.** Random forest analysis sample-based regression performed on survival data showing a variant allele frequency (VAF) cutoff of 23% in *TP53^MT^* as the threshold separating probable monoallelic from probable biallelic mutations. *TP53^MT^*: *TP53* mutation.

**Supplementary Figure 9.** Kaplan-Meier survival estimates comparing groups according to new classification.

Kaplan-Meier survival estimates comparing probable monoallelic, probable biallelic, and obligatory biallelic groups at different vaf cutoffs (12,17,23,37). Mono: probable monoallelic, PBi: probable biallelic, Bi: obligatory biallelic.

**Supplementary Figure 10. Kaplan-Meier survival estimates comparing single and double hits *TP53* groups in MDS and AML patients.** Kaplan-Meier survival estimates comparing single and double hits groups in MDS and AML patients according to our new classifications model separating obligatory biallelic, probable biallelic, and probable monoallelic mutations. MDS: myelodysplastic syndrome, AML, acute myeloid leukemia.

**Supplementary Figure 11. Kaplan-Meier survival estimates of the validation cohort.** Kaplan-Meier survival estimates of validation cohort comparing obligatory biallelic, probable biallelic, and probable monoallelic mutations.

**Supplementary Figure-12** TP53 hits reclassification based on novel method. Flow diagram reclassifying traditionally classified TP53 hits according to our novel method.

**Supplementary Figure 13. Single-cell DNA sequencing in selected samples.** Application of single-cell DNA analysis on selected cases to investigate the *TP53* allelic inactivation. **A.** **Case** UPN13: pAML patient with a single *TP53* N196D mutation (VAF 7.6%) and no chromosome 17 alterations. Single-cell DNA analysis showed that 6.1% of *TP53* mutant cells are actually biallelic. **B.** UPN125: Patient with hypocellular MDS with a single *TP53* C203F mutation (VAF 33%) and chromosome 17p13 addition. Single-cell DNA analysis showed that 4.0% of *TP53* mutant cells were actually biallelic. **C.** UPN423: Pateint with MDS/MPN with two *TP53* missense mutations: R136H and H175P (sum of VAFs 20.1%). Single-cell DNA analysis showed that 32.1% of *TP53* mutant cells were biallelic. **D.** UPN875: Patient with LR-MDS with two *TP53* missense and truncating mutations: V177M and S144* (sum of VAFs 12.7%). Single-cell DNA analysis showed that 34.1% of *TP53* mutant cells were biallelic. Single-cell DNA analysis also identified a third *TP53* mutation that was not detected by NGS bulk sequencing: S222fs in 15 cells.

NGS: next generation sequencing, VAF: variant allele frequency, pAML: primary acute myeloid leukemia, MDS: myelodysplastic syndrome, MDS/MPN: myelodysplastic syndrome/myeloproliferative neoplasm.

**Supplementary Figure 14. Somatic co-mutations in different disease subtypes.** The frequency of non-*TP53* somatic co-mutations per patient in each disease subtype. pAML: primary acute myeloid leukemia, sAML: secondary acute myeloid leukemia, HR-MDS: high-risk myelodysplastic syndrome, LR-MDS: low-risk myelodysplastic syndrome, MDS/MPN: myelodysplastic/myeloproliferative neoplasm.

**Supplementary Figure 15.** **Frequency of concurrent somatic mutations.** Frequency of non-*TP53* somatic co-mutations among AML and MDS patients according to the *TP53* status (*TP53^MT^* *vs. TP53^WT^*) and the *TP53* allelic configurations (obligatory biallelic *vs.* non-obligatory biallelic). AML: acute myeloid leukemia, MDS: myelodysplastic syndrome, *TP53^MT^*: *TP53* mutation, *TP53^WT^*, *TP53* wild-type

**Supplementary Methods**

**Genetic studies**

*For the data collected at CCF, whole-exome sequencing (WES) was performed and paired disease and germline DNA of purified CD3+ lymphocytes were used for 155 samples. Whole-exome capture (n=155) was accomplished by hybridizing sonicated genomic DNAs to a bait cDNA library synthesized on magnetic beads (SureSelect Human All Exon 50Mb or V4 kit, Agilent Technologies). Captured targets were sequenced using a HiSeq 2000 (Illumina) and standard protocols for 100-bp paired-end reads. Reads were aligned to the human genome (hg19) by a Burrows-Wheeler aligner (http://bio-bwa.sourceforge.net/) using a GATK pipeline that also extracted candidate variants/polymorphisms to reduce sequencing errors. Data were validated using targeted sequencing. Targeted sequencing was performed using Illuminas TruSeq (n=1,202) and Nextera Custom Amplicon kit; for this technology an overlapping set of 46 genes was interrogated (Supplementary Table 5). Sequencing libraries were generated according to an Illumina paired-end library protocol. The enriched targets were sequenced using a HiSeq 2000 or MiSeq (Illumina), at 862x coverage. Variants were annotated using Annovar^1^ and filtered by removing: i) synonymous single nucleotide variants; ii) variants only present in 140 unidirectional reads; and iii) variants in repetitive genomic regions. Variants with minimum depth less than 20 or number of high-quality reads less than 5 were filtered out. A bio-analytic pipeline developed in-house^1-3^ was applied to call somatic mutations by cross-match the calls with the ones reported in control databases such as dbSNP138, 1000 Genome Project or ESP 6500, Exome Aggregation Consortium (ExAC) and gnomAD. Variant allele frequencies (VAFs) were adjusted according to zygosity and copy number based on conventional metaphase karyotyping and/or SNP-array results.” Specimens of patients from the MLL cohort were subjected to NGS using different methods and gene panels as previously described^1,2,4^. The gene sequencing methods of publicly-shared data were previously described*^5-8^.

**Conventional cytogenetics**

Metaphase cytogenetics was performed on bone marrow aspirates. The median number of metaphases analyzed was 20. Chromosomal preparation was performed on G-banded metaphase cells using standard techniques, and karyotypes were described for all the patients according to the International System for Human Cytogenetic Nomenclature^9,10^.

**Single-cell DNA analysis**

Bone marrow mononuclear cells from patients with *TP53*mutations (n=4) were thawed overnight in Iscove Modified Dulbecco Media (Thermo Fisher Scientific, Waltham, MA) supplemented with 10% fetal bovine serum. The day after, the cells were washed with 1X phosphate-buffer saline and 3-4,000 cells/µL were resuspended in Tapestri’s cell buffer and encapsulated using Tapestri microfluidics cartridges, lysed, and barcoded using a Mission Bio (South San Francisco, CA) [[https://missionbio.com/](https://protect-us.mimecast.com/s/07XCCW6vMAtjEpx2ki60PF5?domain=missionbio.com/)] sequencer for single-cell DNA analysis. Samples were amplified using a modified 45-myeloid gene panel (Tapestri) including copy number for chromosome 5, 6, 7, and 17. PCR products were purified using Ampure XP beads (Beckman Coulter, Pasadena, CA), quantified for size and concentration using an Agilent bioanalyzer and pooled libraries were run on an Illumina NovaSeq at the genomic core of the Lerner Research Institute (Cleveland Clinic). Variants analysis was conducted using Tapestri Insights platform version 2.2. To differentiate high-quality variants from low-quality variants, a series of metrics were applied. Potential false positive were excluded if: a) relatively a low percentage of genotyped cells per sample was observed; b) comparable number of mutated cells were found across unrelated and independent samples; c) VAF by read count versus VAF by cell count was more than 1.5 fold discrepant; d) average clone and variant specific VAFs deviate from associated zygosity values.

**Statistical methods**

Patients’ characteristics were summarized by cross-tabulations for categorical variables or by quantiles for continuous variables. Fisher’s exact test and Chi-square test were used to compare categorical variables. Mann–Whitney U test/ Wilcoxon rank-sum test were used for continuous variables. All p- values were two-sided; those less than 0.05 were considered statistically significant. All statistical computations were performed using R 3.6.2 (www.r-project.org) and Prism (GraphPad). Overall survival was defined as the time elapsed from disease onset to last follow-up or death. To assess prognostic differences among the identified clusters, pairwise survival analysis using Kaplan Meier estimator and the Log-rank test was performed.

We used random-forest analysis to identify the optima VAF cutoff to determine the allelic status. RandomForestSRC package implements the model which adapts the Classification and Regression Tree (CART) to right-censored survival data optimized over ensemble cumulative hazard function (CHF). CHF for a given terminal node is estimated using the Nelson-Aalen estimator. Over 1000 trees built on random subsamples of input data, we extracted the most common VAF thresholds chosen by the method as the threshold value. A similar VAF threshold value was also reached with ROC analysis. Furthermore, to statistically test whether the identified VAF threshold is informative, we used the Kaplan-Meier estimator and Log-rank test for hypothesis testing (further details in **Supplementary Methods**). In addition, we randomly divided the data into test/train sets with %20/%80 ratios and calculated the Harrell’s C-index (Concordance-index) in the test set over 30 runs.

Our model was validated on an external cohort of 92 patients from University of Southwestern Medical Center and Wayne State University.

**References**

1. Meggendorfer M, Haferlach C, Kern W, Haferlach T. Molecular analysis of myelodysplastic syndrome with isolated deletion of the long arm of chromosome 5 reveals a specific spectrum of molecular mutations with prognostic impact: a study on 123 patients and 27 genes. *Haematologica*. Sep 2017;102(9):1502-1510. doi:10.3324/haematol.2017.166173

2. Delic S, Rose D, Kern W, et al. Application of an NGS-based 28-gene panel in myeloproliferative neoplasms reveals distinct mutation patterns in essential thrombocythaemia, primary myelofibrosis and polycythaemia vera. *Br J Haematol*. Nov 2016;175(3):419-426. doi:10.1111/bjh.14269

3. Haferlach T, Nagata Y, Grossmann V, et al. Landscape of genetic lesions in 944 patients with myelodysplastic syndromes. *Leukemia*. Feb 2014;28(2):241-7. doi:10.1038/leu.2013.336

4. Palomo L, Meggendorfer M, Hutter S, et al. Molecular landscape and clonal architecture of adult myelodysplastic/myeloproliferative neoplasms. *Blood*. Oct 15 2020;136(16):1851-1862. doi:10.1182/blood.2019004229

5. Bersanelli M, Travaglino E, Meggendorfer M, et al. Classification and Personalized Prognostic Assessment on the Basis of Clinical and Genomic Features in Myelodysplastic Syndromes. *J Clin Oncol*. Apr 10 2021;39(11):1223-1233. doi:10.1200/JCO.20.01659

6. Nagata Y, Zhao R, Awada H, et al. Machine learning demonstrates that somatic mutations imprint invariant morphologic features in myelodysplastic syndromes. *Blood*. Nov 12 2020;136(20):2249-2262. doi:10.1182/blood.2020005488

7. Radakovich N, Nagy M, Nazha A. Machine learning in haematological malignancies. *Lancet Haematol*. Jul 2020;7(7):e541-e550. doi:10.1016/S2352-3026(20)30121-6

8. Tyner JW, Tognon CE, Bottomly D, et al. Functional genomic landscape of acute myeloid leukaemia. *Nature*. Oct 2018;562(7728):526-531. doi:10.1038/s41586-018-0623-z

9. Gonzalez Garcia JR, Meza-Espinoza JP. Use of the International System for Human Cytogenetic Nomenclature (ISCN). *Blood*. Dec 1 2006;108(12):3952-3; author reply 3953. doi:10.1182/blood-2006-06-031351

10. Liehr T. International System for Human Cytogenetic or Cytogenomic Nomenclature (ISCN): Some Thoughts. *Cytogenet Genome Res*. 2021;161(5):223-224. doi:10.1159/000516654
